# Gold Nanoparticles Decorated with Mannose-6-phosphate Analogues

**DOI:** 10.3390/molecules19011120

**Published:** 2014-01-17

**Authors:** Stéphanie Combemale, Jean-Norbert Assam-Evoung, Sabrina Houaidji, Rashda Bibi, Véronique Barragan-Montero

**Affiliations:** Institut des Biomolécules Max Mousseron UMR 5247 UM2-UM1-CNRS-ENSCM 8 rue de l’Ecole Normale, Montpellier cedex 5 34296, France

**Keywords:** C-C coupling reaction, click chemistry, glycoconjugate, mannose-6-phosphate, glyconanoparticle, angiogenesis

## Abstract

Herein, the preparation of neoglycoconjugates bearing mannose-6-phosphate analogues is described by: (a) synthesis of a cyclic sulfate precursor to access the carbohydrate head-group by nucleophilic displacement with an appropriate nucleophile; (b) introduction of spacers on the mannose-6-phosphate analogues via Huisgen’s cycloaddition, the Julia reaction, or the thiol-ene reaction under ultrasound activation. With the resulting compounds in hand, gold nanoparticles could be functionalized with various carbohydrate derivatives (glycoconjugates) and then tested for angiogenic activity. It was observed that the length and flexibility of the spacer separating the sugar analogue from the nanoparticle have little influence on the biological response. One particular nanoparticle system substantially inhibits blood vessel growth in contrast to activation by the corresponding monomeric glycoconjugate, thereby demonstrating the importance of multivalency in angiogenic activity.

## 1. Introduction

The ensuing article was initially motivated by the biomedical importance of mannose 6-phosphate (M6P) [1]. While two different mannose 6-phosphate receptors (M6PR) recognize the M6P residues and mediate the endocytosis of extracellular M6P-containing ligands, only the larger of these (CI-M6PR, 275 kDa) has been reported to also bind retinoic acid and IGF-II [2]. The biological importance of this receptor is found in numerous processes and it has been reported that the angiogenic action of proliferin was mediated by this receptor [3]. We have recently described the synthesis of a series of mannose-6-phosphate (M6P) analogues, showing for the first time that these monosaccharides play a role in angiogenesis [4,5,6,7]. The replacement of the phosphate head-group by analogues, mostly bioisosteres, was intended to provide a better understanding of the chemical factors involved in the modulation of angiogenic activities. It is known, however, that a monovalent carbohydrate ligand possesses only a weak binding affinity toward its associated receptor protein [8,9,10]. To impart biological relevance to such interactions Nature often utilizes multivalency [11,12]. Therefore, interest in designing multivalent carbohydrate systems has been growing [13]. In particular, glyconanoparticles (GNPs), that offer useful tools for investigating carbohydrate-mediated interactions, have been developed [14]. The purpose of the present study was: (a) to synthesize new glycoconjugates bearing M6P-like groups and (b) to insert these compounds onto the surfaces of gold particles via a spacer for angiogenic testing. Our objective, therefore, is to investigate the effect of clustered sugar derivatives on angiogenesis and to determine whether or not the spacer has an influence on the biological response. The choice of the M6P analogues has been guided by previous results conducted in our laboratory including the synthesis of carboxylate and azido analogues (with 123% and 125% relative angiogenic activity, respectively, compared to phosphate buffer saline (PBS) as control in an egg membrane assay) [4]. Additional considerations include varying the length, hydrophilic or hydrophobic nature, and flexibility of the spacer between the sugar headgroups and the nanoparticle core. In this manner we could modify the presentation of the carbohydrates and, consequently, affect their accessibility during the molecular recognition events. Many of the mannose derivatives with their different spacers were assembled using the “click chemistry” strategy introduced by Huisgen and improved by Sharpless and co-workers in 2001 [15,16]. Within a short time-frame, the click chemistry reaction has proven to be of remarkable utility and broad scope, not only in organic synthesis but in chemical biology and drug discovery [17,18]. Although 1,3-dipo lar cycloaddition reaction is central to click chemistry, the resulting creation of a triazole moiety may have an adverse influence on a biological response. For this reason two other reactions were used for chain elongation or for conjugation of two synthons: the Julia reaction and the thiol-ene reaction that was run under unprecedented ultrasound activation.

## 2. Results and Discussion

The preparation of the neoglycoconjugates we describe herein took place in three major steps: (a) the synthesis of a cyclic sulfate precursor to access the ligand head-group by nucleophilic displacement with the appropriate nucleophile; (b) the introduction of the spacers on the M6P via either Huisgen’s cycloaddition, the Julia reaction, or the thiol-ene reaction under ultrasound activation; (c) the coupling between the spacer and the sugar moiety. By this means gold nanoparticles as functionalized by various carbohydrates could be compared for their effect on angiogenic processes. Although preliminary biological data are presented at the end of the paper, the emphasis here will be on the synthetic challenges involved in obtaining the necessary neoglycoconjugates.

### 2.1. 1,3Dipolar Cycloaddition

**Scheme 1:**
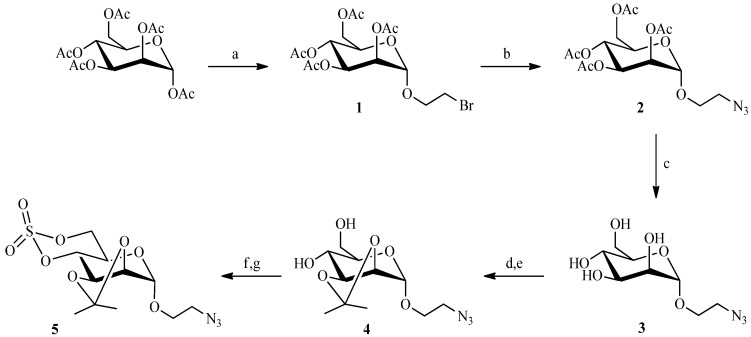
Preparation of the cyclic sulfate intermediate **5**.

Huisgen’s 1,3-dipolar cycloaddition is the primary example of a “click reaction”. It is the reaction between a 1,3-dipole (an azide) and a dipolarophile (an acetylene) to form a five-membered heterocycle. The classical reaction proceeds by a concerted mechanism under thermal conditions to afford a mixture of 1,4- and 1,5-disubstituted [1,2,3]-triazole regioisomers [19], but when the reaction is catalyzed by Cu(I), only the 1,4-substituted-triazole is obtained [20]. We selected this reaction as one means for securing our mannose-6-phosphate analogues. Thus to prepare the nanoparticles, the carbohydrate moiety had to bear either an azide or alkyne function. The linker chain, in turn, would provide the complementary group. The cyclic sulfate strategy, utilized in our laboratory to prepare M6P analogues, demanded that the carbohydrate possess the azide group because an alkyne function would become oxidized during the preparation of the sulfate. Thus, peracetylated mannose has been coupled in very good yield to 2-bromoethanol, under classical conditions [21], in the presence of boron trifluoride etherate (Scheme 1). The azide group was then introduced with sodium azide, and the acetate protecting groups were removed under Zemplen conditions [22] to give the 2-azidoethyl-α-d-mannopyranoside **3**. After selectively introducing isopropylidene protection at the 2 and 3 positions of the mannose, the cyclic sulfate **5** was prepared according to a modified published procedure [23,24]. Compound **4** was converted via thionyl chloride into the cyclic sulfite which was then oxidized by ruthenium oxide (prepared *in situ*) into the corresponding cyclic sulfate **5**.

*Reagents and conditions*: (a) 2-bromoethanol, BF_3_·OEt_2_, CH_2_Cl_2_, 91%; (b) NaN_3_, DMF, 96%; (c) MeONa, MeOH, 65%; (d) 2,2 dimethoxypropane, APTS, acetone; (e) AcOH, H_2_O, 85%; (f) SOCl_2_, NEt_3_, CH_2_Cl_2_; (g) RuCl_3_, NaIO_4_, CH_2_Cl_2_/CH_3_CN, 66%.

**Scheme 2:**
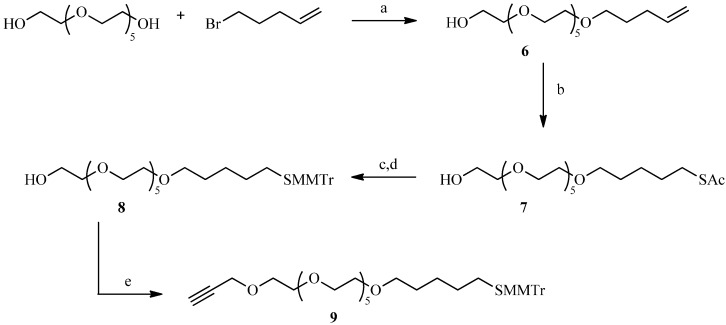
Preparation of the alkyne **9** for the click reaction.

A “spacer” refers to a chain that can be used to join our sugar derivatives to the gold particles. One of the spacers, possessing an alkyne unit for reaction with a sugar-azide, was designed on the basis of its flexibility and aqueous solubility (Scheme 2). Thus, the reaction of 5-bromopentene with a slight excess of 50% sodium hydroxide and hexaethylene glycol provided the monoether **6** [25]. Photochemical addition of thioacetic acid to the double bond gave the thioacetate in good yield [26]. The next step was to introduce the alkyne function on the spacer in the presence of NaH, but the acetate protecting groups, being sensitive to hydrides, were first replaced by 4-methoxytrityl. Thus, compound **7** was deacetylated by concentrated hydrochloric acid in ethanol to avoid the formation of disulfide under basic conditions. The thiol was then protected by reaction with 4-methoxytrityl chloride. Finally, the free hydroxyl of **8** was reacted with 3-bromopropyne in the presence of sodium hydride in anhydrous THF to introduce the alkyne function required for the click reaction. 

*Reagents and conditions*: (a) NaOH 50%, 99%; (b) AcSH, THF, 71%; (c) Conc. HCl, EtOH; (d) MeOTrCl, THF, 91%; (e) 2-bromopropyne, NaH, THF, 97%.

**Scheme 3:**
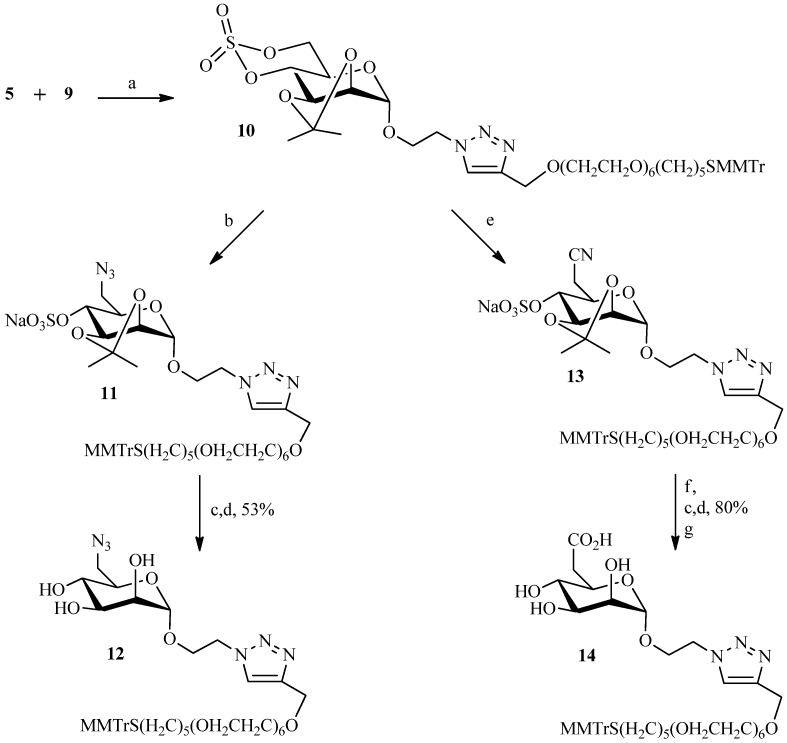
Preparation of the functionalized ligands **12** and **14**.

The literature describes a variety of ways in which the Huisgen cycloaddition can be performed to join two entities. Sources of copper (I) catalyst can be produced *in situ* by reduction of copper (II) salts [20] or obtained through disproportionation of Cu (0) and Cu (II) salts [27]. Cu (I) can also be introduced as copper (I) salts such as CuI or obtained from oxidation of Cu (0) salt [28,29,30,31]. In search for the optimal reaction conditions, we initially tested the most commonly employed system, namely CuSO_4_·5H_2_O and sodium ascorbate as source of copper (I) in *tert*-BuOH/H_2_O [32]. Interestingly, no reaction was observed after 24 h. In addition, several parameters were altered without success: increasing the concentration of reactants, changing the ratio copper/sodium ascorbate, using a co-solvent (acetonitrile), or substituting tBuOH with pyridine. The click reaction was also attempted using cuprous iodide in pyridine as catalyst [33]. Despite many modifications to the original protocol the desired product was never obtained in good yield. Thus, another copper catalyst system consisting of formation of Cu(I) by oxidation of copper metal was investigated. The oxidative cycloaddition of Cu(0) with ammonium chloride [34] in a mixture of *tert*-BuOH/H_2_O was also unsuccessful. It should be noted that heating to 40–60 °C, and increasing the reagents’concentration, failed to improve the performance, as they often do in many examples of click chemistry reactions, but led only to degradation. Ultrasound in place of classical activation was carried out again without success. Only a system using copper powder, rarely encountered in the literature, gave positive results, giving compound **10** in 60% yield (Scheme 3). Starting from compound **10** two mannose-6-phosphate analogues were prepared with only slight modification to the previously reported protocols [4]. First, the azide function was easily introduced on the cyclic sulfate **10** by reaction with sodium azide to afford compound **11**. Although isopropylidene and trityl are usually deprotected under acid conditions, our assays did not allow simultaneous cleavage of the two functions. The final ligand **12** was therefore obtained in two separate steps. The trityl group was first cleaved by ceric ammonium nitrate (a redox reaction) [35,36] prior to deprotection of the isopropylidene and the sulfate groups via acidic ion exchange resins. To afford the carboxylic acid analogue of M6P **14**, sodium cyanide was first reacted with the cyclic sulfate **10**, and the nitrile function was then hydrolyzed with sodium hydroxide in a 30% solution of hydrogen peroxide to give the corresponding carboxylic acid. The ligand **14** was obtained using the same deprotection conditions as described for the azide analogue **12** (Scheme 3).

*Reagents and conditions*: (a) Cu(I), NHEt_3_Cl, tBuOH/H_2_O, 60%; (b) NaN_3_, DMF, 62%; (c) CAN, CH_3_CN/H_2_O; (d) Amberlyst H^+^, MeOH/THF; (e) NaCN, DMF, 65%; (f) H_2_O_2_, NaOH, 52%; (g) AcOH, H_2_O, 78%.

### 2.2. Julia Reaction

Among the olefination reactions to form a regio- and stereoselective alkene, the Julia olefination is one of the well-known methods, along with the Wittig reaction [37,38], the Wittig-Horner reaction [39,40,41], the Horner-Wadsworth-Emmons [42,43], the Peterson reaction [44,45,46] and the Johnson reaction [47]. The classical Julia olefination, also known as the Julia-Lythgoe olefination, was developed fourty years ago and is based on a reductive elimination process of β-acyloxysulfones [48]. Since its discovery, significant improvements have been made to the methodology of this reaction, and it has become a crucial step in the synthesis of many natural products. A new variant of the classical Julia reaction, the Julia-Kocienski olefination, also called modified or one-pot Julia olefination, has recently emerged as a powerful tool for olefin synthesis [49,50,51]. The process involves the replacement of the aryl sulfone moiety, traditionally used in the classical reaction, with different heteroaryl sulfones, thus allowing a direct olefination process.

**Scheme 4:**
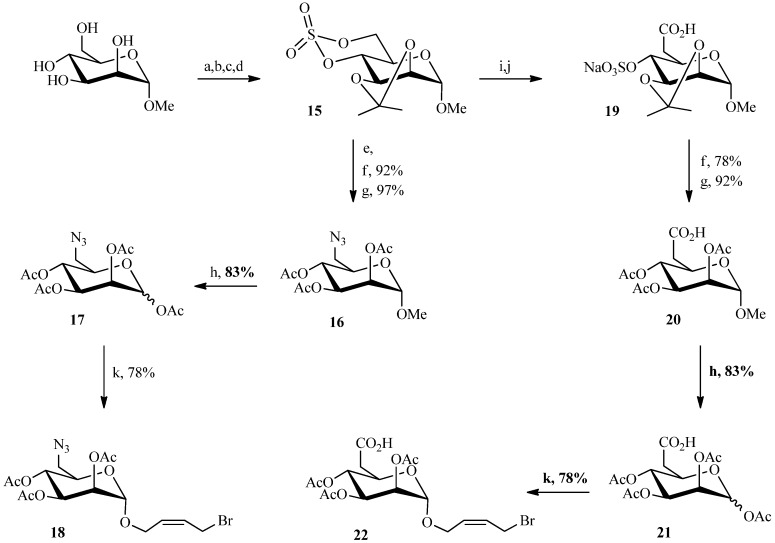
Preparation of the allyl bromides **18** and **22**.

In our Julia olefination, a carbohydrate block was derivatized with an allyl bromide function (to be later joined with a sulfone-bearing linker). The initial steps in the sugar portion of the molecule followed the same strategy as described for compound **5** (Scheme 4). The methyl α-d-mannopyranoside was previously protected with two O-isopropylidene groups on the 2,3 and 4,6 positions using 2,2-dimethoxypropane and *para*-toluenesulfonic acid. After the selective opening of isopropylidene at the 4,6 positions with a mixture of AcOH/H_2_O, the cyclic sulfite was obtained by reaction with thionyl chloride and triethylamine, and subsequent oxidation afforded the cyclic sulfate **15** in good yield. In contrast to the chemistry in Scheme 1 and Scheme 3, the azide and carboxylic acid analogues of M6P were prepared prior to the coupling reaction. Therefore, sodium azide was reacted with the cyclic sulfate **15** to give compound **16**. A solution of acetic acid in water led to the cleavage of the isopropylidene and the sulfate. The replacement of the anomeric methyl group by an acetyl group led to compound **17** in 83% yield. The allyl bromide unit required to perform the coupling reaction was then introduced by glycosylation with *cis*-1-bromo-but-2-en-4-ol. The same strategy was applied to form the carboxylic acid analogue of M6P.

*Reagents and conditions*: (a) 2,2-dimethoxypropane, APTS, acetone; (b) AcOH/H_2_O, 63%; (c) SOCl_2_, NEt_3_, CH_2_Cl_2_; (d) RuCl_3_, NaIO_4_, CH_2_Cl_2_/CH_3_CN, 85%; (e) NaN_3_, DMF, 92%; (f) AcOH/H_2_O; (g) Ac_2_O, DMAP, pyridine; (h) AcOH, H_2_SO_4_, Ac_2_O; (i) NaCN, DMF, 100%; (j) H_2_O_2_, NaOH, 100%; (k) HOCH_2_CH=CHCH_2_Br, BF_3_·OEt_2_, CH_2_Cl_2_, 78%.

**Scheme 5:**
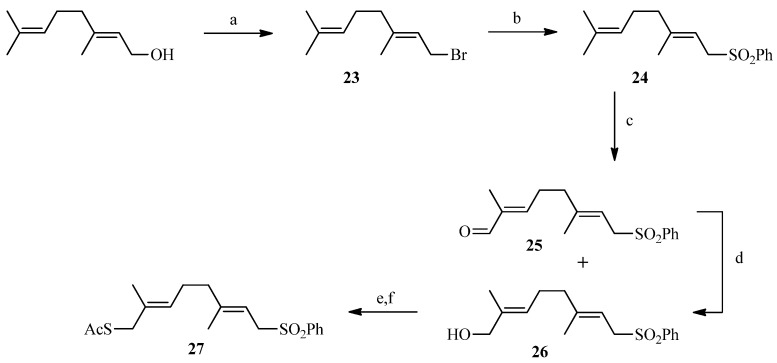
Synthesis of the sulphone **27**.

**Scheme 6:**
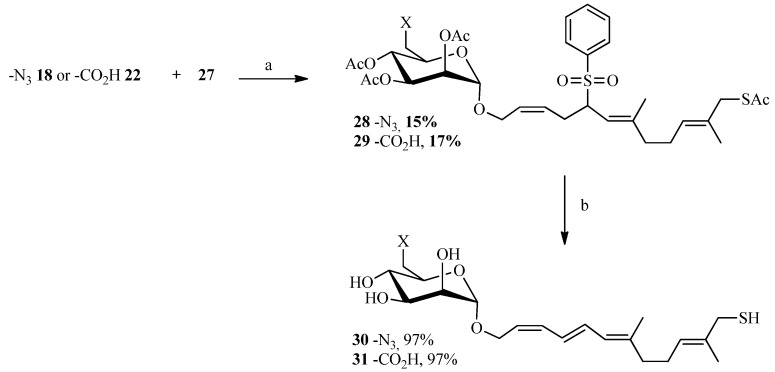
Coupling reaction between the allyl bromides and the sulphone.

To create the Julia spacer (Scheme 5), geraniol was reacted with phosphorus tribromide to give geranyl bromide **23** in good yield followed by a reaction with sodium phenylsulfinate to provide the desired sulfone **24**. Next, functionalization of the other side of the linker was carried out in one step via oxidation of a terminal methyl by selenium dioxide. The strategy described by Sharpless using *tert*-butyl hydroperoxide as an oxidant [52] was utilized: **24** was reacted with a catalytic amount of selenium oxide in the presence of *tert*-butyl hydroperoxide which enables the recycling of selenium dioxide. A 50/50 mixture of alcohol and aldehyde was obtained and, after purification, the aldehyde was reduced with sodium borohydride to give compound **26** in 63% yield. The alcohol **26** was then brominated with tetrabromomethane and triphenylphosphine after which the thiol group was introduced by reaction with potassium thioacetate. A “click-type” reaction was then performed between **18** or **22** and **27** in the presence of lithium bis(trimethylsilyl)amide (LiHMDS) in THF from which compounds **28** and **29** were obtained in 15% and 17% yield, respectively. Deprotection of the acetates and removal of the sulfone group under basic conditions gave the desired final compounds **30** and **31** in almost quantitative yield (Scheme 6). The linker in this case is polyunsaturated.

*Reagents and conditions*: (a) PBr_3_, CH_2_Cl_2_, 93%; (b) NaSO_2_Ph, DMF, 93%; (c) SeO_2_, tBuOOH, HOPhCO_2_H, CH_2_Cl_2_; (d) NaBH_4_, MeOH, 63% of 26; (e) CBr_4_, PPh_3_, CH_2_Cl_2_, 100%; (f) KSAc, DMF, 100%.

*Reagents and conditions*: (a) LiHMDS, THF; (b) MeONa, MeOH.

### 2.3. Thiol-ene Reaction

**Scheme 7:**
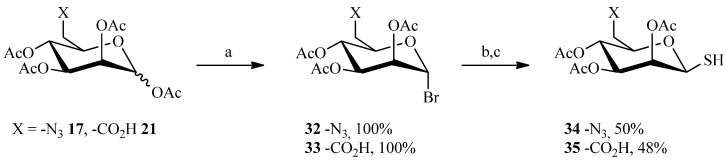
Preparation of the thiosugars **34** and **35**.

One reaction that is emerging as an attractive click-type process is the century-old addition of thiols to alkenes [53], which is currently called thiol-ene coupling. In fact, the thiol-ene reaction is simply the hydrothiolation of a C=C bond, and proceeds by a radical mechanism, induced photochemical or thermally, to give an *anti*-Markovnikov-type thioether [54,55]. The reaction discovered in 1905 by Posner [56] has been widely used in the mid-nineteenth century, especially in polymer chemistry. However, the thiol-ene reaction has recently attracted researchers in other areas of synthesis due to recognition of its ‘‘click-type’’ characteristics: highly efficient and orthogonal to a wide range of functional groups, as well as compatible with water and oxygen. Thus, the thiol-ene reaction enables the establishment of a rapid ligation between two entities assisted by the stability of the thioether linkage in a wide range of chemical environments. To perform the reaction, the thiol function was placed on the sugar moiety while the spacer carried the vinylic group. As before, the M6P derivatives were prepared using the cyclic sulfate strategy prior to performing the click-style reaction (Scheme 7). The synthesis began by replacing the anomeric acetate with bromine on compounds **17** and **21** described previously. This was accomplished with a solution of hydrobromic acid/acetic acid in quantitative yields. The thiol function was then introduced in two steps, first via thiourea in acetone then removal of the nitrogens with sodium metabisulfide. Only thiosugars (**34** and **35**) having the β configuration were obtained.

*Reagents and conditions*: (a) HBr/AcOH, Ac_2_O; (b) thiourea, Acetone; (c) Na_2_S_2_O_5_, CHCl_3_/H_2_O.

**Scheme 8:**
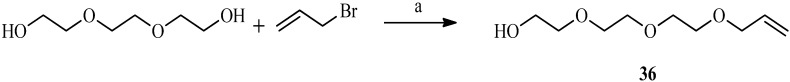
Preparation of the spacer.

Having chosen to synthesize a fully flexible spacer (Scheme 8), the triethylene glycol was coupled to allyl bromide in the presence of 50% aqueous sodium hydroxide.

Reagents and conditions: (a) NaOH 50%, 85%.

**Scheme 9:**
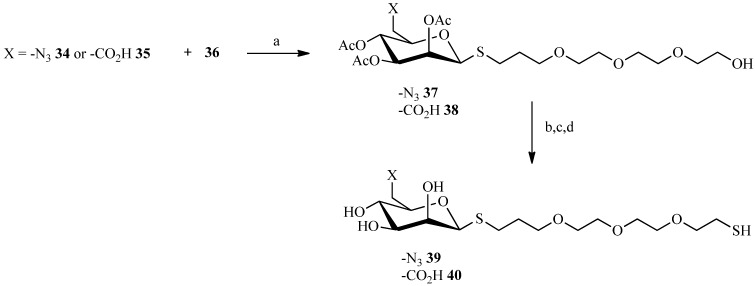
Preparation of the thiosugars **39** and **40** by click thiol-ene reaction.

Then, to facilitate the reaction, and to avoid formation of byproducts, the free hydroxyl of the linker was brominated and thioacetylated only at the end of the synthesis. Actually, coupling can be accomplished between a protected thiol group and an alkene or between a thiol and an alkene. However, the final thioether was obtained with better results when the anomeric thiol was not protected (Scheme 9). The initiation step can be triggered in several ways, by simple heating or by ultraviolet irradiation. Another method of initiation has been developed in the laboratory which is to perform the coupling under ultrasound (Table 1). When THF was replaced by dioxane, yields increased by 10%.

*Reagents and conditions*: (a) AIBN, dioxane, US activation, 80%; (b) CBr_4_, PPh_3_, CH_2_Cl_2_; (c) KSAc, DMF; (d) MeONa, MeOH.

**Table 1:** Comparative results for the click thiol-ene reaction.

Compound	Reflux, THF, 24 h	UV, THF, 5 h	Sonication, THF, 4 h	Sonication, Dioxane, 3 h
**39**	76%	50%	72%	79%
**40**	78%	60%	75%	80%

### 2.4. Gold Nanoparticles

Research in developing new synthesis protocols to generate gold nanoparticles (AuNPs) with desired properties has received immense attention due to their considerable applications in biomedical field [57]. One of the primary prerequisites for using AuNPs in biomedical application is that they are non-toxic and biocompatible to both *in vitro* and *in vivo* environments. Secondly, AuNPs should be coated with a protective layer to prevent aggregation. Thirdly, AuNPs need to be labeled with biologically relevant biomolecules to impart specificity for their potential application. The two most interesting and common methods to prepare AuNPs are the Brust method [58] utilizing NaBH_4_ (which can’t be used in our case because NaBH_4_ would reduce the azide function of our derivatives) and the citrate method. This latter method includes only three starting materials, namely, auric acid, sodium citrate (the reducer), and water. Following a report by Turkevich *et al.* in 1951 [59], this synthetic scheme has been widely studied and often used for the preparation of AuNPs-based materials [60,61,62,63]. We have developed a protocol by adjusting the gold-to-citrate ratio to obtain 10 nm AuNPs (Table 2). Details are given in the Experimental section.

**Table 2:** Size of the nanoparticles in nm.

Nanoparticles	TEM ^[a]^	DLS ^[b]^
Azide AuNPs (Huisgen)	10	18–20
Azide AuNPs (Julia)	10	14–16
Azide AuNPs (thiol-ene)	10	12–13
Carboxylic acid AuNPs (Huisgen)	10	19–20
Carboxylic acid AuNPs (Julia)	10	15–16

[a] Transmission electron microscopy [b] Dynamic light scattering.

### 2.5. Biological Assays

**Figure 1:**
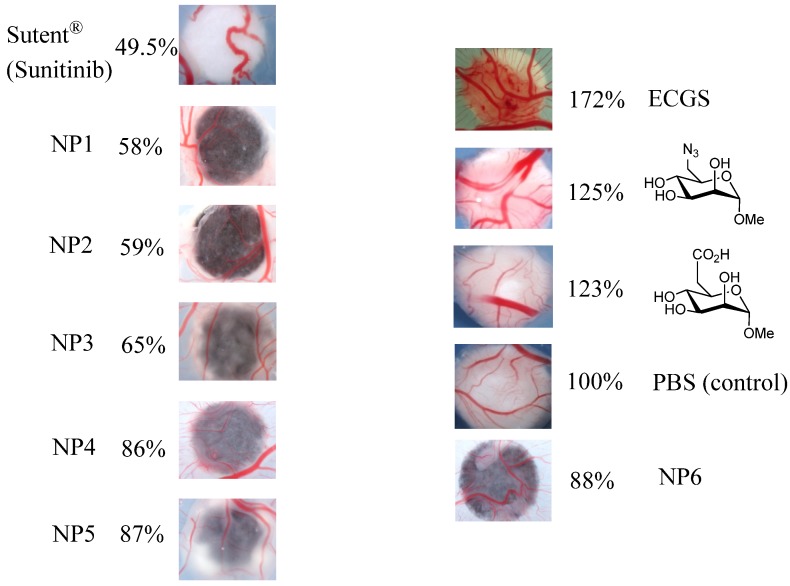
CAM assays using gold nanoparticles functionalized with mannose-6-phosphate analogues compared to angiogenic inhibitor Sutent^®^, angiogenic activator Endothelial Cell Growth Supplement (ECGS) and Phosphate Buffer Saline (PBS) as control.

AuNPs functionalized with M6P analogues have been subjected to angiogenic assays using an experimental model, the avian chorioallantoic membrane assay (CAM) [64,65,66]. Paper discs were saturated with a phosphate buffer saline dispersion of coated AuNPs (60 mg/mL) in PBS or a control (phosphate buffer saline) and then deposited on chorioallantoic membranes of 7-day-old chicken embryos for 4 days in ovo at 38 °C. Sutent^®^ (sunitinib, a non-proteic inhibitor) and endothelial cell growth supplement (ECGS) were used at 60 mg/mL as negative and positive stimuli, respectively. Quantification of the angiogenic response was carried out by measuring the area of neo-vascularization on each particular membrane (Figure 1) using Image J software. The experiments have been repeated at least four times, and the results were reproducible (see experimental part for details).

*Nanoparticles*: NP1: azide-AuNPs thiol-ene; NP2: azide-AuNPs Julia; NP3: azide-AuNPs Huisgen; NP4: Carboxylic-AuNPs Huisgen; NP5: Carboxylic-AuNPs Julia; NP6: Carboxylic-AuNPs thiol-ene.

These experiments demonstrate that all our prepared AuNPs are CAM-inhibitors. Study of the three azide-AuNPs synthesized according to the coupling methods (NP1: thiol-ene 58%, NP2: Julia 59%, NP3: Huisgen 65%) revealed that the length and flexibility of spacers have little influence on the observed biological response. Interestingly, the azide sugar-monomer is a good angiogenic activator (125%), whereas the functionalized -N_3_ nanoparticles, representing a multi-valent collection of sugars, show a strong inhibitory effect (58%–65%). Similar results were obtained for the three carboxylic acid-AuNPs (NP4: Huisgen 86%, NP5: Julia 87%, NP6: thiol-ene 88%). Comparison of the activating effect of the carboxylic acid analogue (123% observed in previous work) [4] and the inhibitory effect of the carboxylic acid-AuNPs (86% to 88% compared to the control) indicates that multi-valency can do more than qualitatively affect the magnitude of blood vessel formation; it can convert a significant catalyzed process into an inhibition.

## 3. Experimental

### 3.1. General Information

Reactions were monitored by TLC using aluminum-backed plates coated with silica gel 60 F_254_ (Merck); spots were visualized with UV light (254 nm) and/or (a) by staining with *p*-anisaldehyde solution [anisaldehyde (25 mL), H_2_SO_4_ (25 mL), EtOH (450 mL), and CH_3_COOH (1 mL)], followed by heating or (c) by immersion in a 10% H_2_SO_4_/EtOH solution followed by charring. Column chromatography was performed on Carlo-Erba silica gel 60A (35–70 µm). Melting points were determined in capillary with a Büchi melting apparatus 530. Optical rotations were measured at the sodium D-line with a Perkin-Elmer-241 polarimeter. ^1^H-NMR spectra (400.13 MHz) and ^13^C-NMR spectra (100.62 MHz) were recorded on a Bruker DRX 400 instrument. Chemical shifts (δ) are given in parts per million and referenced using residual solvent signals (7.24 ppm for CHCl_3_ and 4.79 ppm for HOD). The following abbreviations were used to explain the signal multiplicities or characteristics: s (singlet), d (doublet), dd (double doublet), ddd (double double doublet), t (triplet), td (triplet doublet), q (quartet), and m (multiplet). Chemical shifts (δ) are given in parts per million relative to TMS as an external reference. Electron ionization mass spectra were recorded in positive or negative mode on a Waters MicroMass/ZQ 2615. Anhydrous solvents were obtained prior to use according to standard methods [67]. For transmission electron microscopy (TEM) examinations, a single drop (10 μL) of an aqueous solution (*ca.* 0.1 mg/mL in Milli-Qwater) of the gold glyconanoparticles (AuNPs) was placed on a coppergrid coated with a carbon film (Electron Microscopy Sciences). The grid was left to dry in air for several hours at room temperature. TEM analysis was performed with a JEOL 1200 EXII microscope, operating at 120 kV. Dynamic Light Scattering (DLS) analyses were performed on a MALVERN HPPS.

*2'-Bromoethyl-2,3,4,6-tetra-O-acetyl-α-d-mannopyranoside* (**1**): To 1,2,3,4,6-penta-*O*-acetyl-α-d-mannopyranoside (540 mg, 1.38 mmol) dissolved in CH_2_Cl_2_ (5 mL) were added 2-bromoethanol (0.2 mL, 2.77 mmol) and BF_3_·Et_2_O (870 µL, 6.92 mmol). After 20 h stirring at room temperature, the mixture was diluted with CH_2_Cl_2_, washed with water, a saturated solution of NaHCO_3_ then water again. The organic layers were combined, dried over Na_2_SO_4_ and concentrated *in vacuo*. Purification by chromatography on silica gel (EtOAc/petroleum ether 1:1) gave the title compound as a white powder (91%). *R*_f_ = 0.86 (EtOAc/toluene 1:1); mp: 116–118 °C (lit. 115–117 °C); 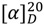 = +42.1 (*c* = 0.5 in chloroform); ^1^H-NMR (CDCl_3_): *δ* = 2.00, 2.05, 2.11, 2.16 (4s, 12H, 4C*H*_3_); 3.52 (t, *J* = 6.0 Hz, 2H, C*H*_2_Br); 3.93 (m, 2H, C*H*_2_CH_2_Br); 4.13 (m, 2H, *H*_5_ and *H*_6a_); 4.27 (dd, 1H, *J* = 5.8 Hz, *J* = 12.6 Hz, *H*_6b_); 4.88 (d, *J* = 1.6 Hz, *H*_1_); 5.27 (dd, 1H, *J* = 2.0 Hz, *J* = 3.2 Hz, *H*_2_); 5.29 (t, 1H, *J* = 1.6 Hz, *H*_4_); 5.35 ppm (dd, 1H, *J* = 3.6 Hz, *J* = 10.0 Hz, *H*_3_);^13^C-NMR (CDCl_3_): *δ* = 20.67, 20.70, 20.75, 20.87 (4*C*H_3_); 29.60 (*C*H_2_CH_2_Br); 62.41 (*C*_6_); 66.00 (C_4_); 68.48 (*C*H_2_Br); 68.93 (*C*_5_); 69.02 (*C*_3_); 69.42 (*C*_2_); 97.75 (*C*_1_); 169.76, 169.86, 170.03, 170.62 ppm (4*C*O); MS (ESI) *m/z*: 477.01, 478.95 [M+Na]^+^.

*2'-Azidoethyl-2,3,4,6-tetra-O-acetyl-α-d-mannopyranoside* (**2**): Sodium azide (1.64 g, 25.05 mmol) was added to a suspension of compound **1** (5.7 g, 12.53 mmol) in DMF (50 mL). After 4 h at 65 °C, the mixture was poured into brine and extracted with CH_2_Cl_2_. The organic extracts were dried (Na_2_SO_4_) and concentrated *in vacuo*. The residue was purified by flash chromatography on silica gel (petroleum ether/EtOAc 4:1) to give the appropriated intermediate as a white solid (96%). *R*_f_ = 0.86 (EtOAc/petroleum ether 1:1); mp: 80–82 °C (lit. 81.8–82.1 °C); 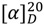 = +39.0 (*c* = 0.6 in chloroform); ^1^H-NMR (CDCl_3_): *δ* = 2.0, 2.05, 2.11, 2.16 (4s, 12H, 4C*H*_3_); 3.47 (m, 2H, C*H*_2_N_3_); 3.67 (m, 1H, C*H*_2_CH_2_N_3_); 3.87 (m, 1H, C*H*_2_CH_2_N_3_); 4.05 (ddd, 1H, *J* = 2.4 Hz, *J* = 5.2 Hz, *J* = 9.7 Hz, *H*_5_); 4.13 (dd, 1H, *J* = 2.6 Hz, *J* = 12.2 Hz, *H*_6a_); 4.29 (dd, 1H, *J* = 5.2 Hz, *J* = 12.4 Hz, *H*_6b_); 4.87 (d, 1H, *J* = 1.6 Hz, *H*_1_); 5.30 (t, 1H, *J* = 10.0 Hz, *H*_4_); 5.28 (dd, 1H, *J* = 2.0 Hz, *J* = 3.2 Hz, *H*_2_); 5.36 ppm (dd, 1H, *J* = 3.2 Hz, *J* = 10.0 Hz, *H*_3_); ^13^C-NMR (CDCl_3_) : δ = 20.63, 20.68, 20.71, 20.84 (4*C*H_3_); 50.32 (*C*H_2_N_3_); 62.42 (*C*_6_); 65.96 (*C*_4_); 67.02 (*C*H_2_CH_2_N_3_); 68.82 (*C*_5_ and *C*_3_); 69.36 (*C*_2_); 97.71 (*C*_1_); 169.73, 169.78, 169.98, 170.59 ppm (4*C*O); MS (ESI) *m/z*: 440.12 [M+Na]^+^.

*2'-Azidoethyl-α-d-mannopyranoside* (**3**): Compound **2** (16.0 g, 38.36 mmol, 1 eq.) and NaOMe (2.07 g, 38.36 mmol, 1 eq.) were added to methanol (100 mL). After 30 min stirring at RT, the mixture was neutralized with Amberlite IRC-50 H^+^ resins, filtered and concentrated *in vacuo*. Purification by chromatography on silica gel (CH_2_Cl_2_/MeOH 9:1) gave a white powder (65%). *R*_f_ = 0.40 (CH_2_Cl_2_/MeOH 4:1); 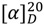 = + 54.9 (*c* = 1.00 in chloroform); ^1^H-NMR (CD_3_OD): *δ* = 3.41 (t, 2H, *J* = 5.0 Hz, C*H*_2_N_3_); 3.60 (m, 3H, *H*_3_, *H*_5_ and C*H*_2_CH_2_N_3_); 3.71 (m, 2H, *H*_4_ and *H*_6a_); 3.85 (m, 2H, *H*_2_ and *H*_6b_); 3.92 (m, 1H, C*H*_2_CH_2_N_3_); 4.81 ppm (d, 1H, *J* = 1.2 Hz, *H*_1_); MS (ESI) *m/z*: 272.11 [M+Na]^+^, 288.02 [M+K]^+^, 521.19 [2M+Na]^+^.

2*'*-Azidoethyl-2,3-*O*-isopropylidene-α-d-mannopyranoside (**4**): A solution of compound **3** (9.5 g, 38.15 mmol, 1 eq.), 2,2-dimethoxypropane (23.4 mL, 190.76 mmol, 5 eq.) and *para*-toluenesulfonic acid (362 mg, 1.90 mmol, 0.05 eq.) in acetone (40 mL) was stirred for 4 h at RT. The *para*-toluenesulfonic acid was neutralized with 5% aq NaHCO_3_. Acetone was removed *in vacuo* and the aqueous phase was washed with petroleum ether to remove the diisopropylidene species. This organic layer was dried (Na_2_SO_4_) and concentrated *in vacuo*. Then the aqueous layer containing the monoisopropylidene was lyophilized. The diisopropylidene compound (7.2 g, 21.88 mmol, 1 eq.) was stirred in a solution of acetic acid/water 8:2 (60 mL) at 35 °C. After 2 h, solvents were evaporated, and then coevaporated with toluene several times. The crude product obtained was purified by chromatography on silica gel (petroleum ether/EtOAc 2:3) to give a yellow oil (85% monoisopropylidene compound, over two steps).

*Diisopropylidene derivative*: *R*_f_ = 0.63 (EtOAc/petroleum ether 1:1); ^1^H-NMR (acetone-*d*_6_): *δ* = 1.31, 1.32 (2s, 6H, 2C*H*_3_); 1.47, 1.48 (2s, 6H, 2C*H*_3_); 3.50 (t, 2H, *J* = 4.8 Hz, C*H*_2_N_3_); 3.53 (m, 1H, *H*_5_); 3.72 (m, 3H, *H*_6a_, *H*_4_ and C*H*_2_CH_2_N_3_); 3.82 (dd, 1H, *J* = 5.8 Hz, *J* = 10.6 Hz, *H*_6b_); 3.93 (qt, 1H, *J* = 5.2 Hz, C*H*_2_CH_2_N_3_); 4.03 (dd, 1H, *J* = 5.6 Hz, *J* = 8.0 Hz, *H*_3_); 4.18 (d, 1H, *J* = 5.6 Hz, *H*_2_); 5.09 (s, 1H, *H*_1_); ^13^C-NMR (acetone-*d*_6_): *δ* = 20.11, 29.38, 27.45, 30.50 (4*C*H_3_); 52.18 (*C*H_2_N_3_); 63.48, 63.53 (*C*_5_ and *C*_6_); 68.17 (*C*H_2_CH_2_N_3_); 74.47 (*C*_4_); 76.78 (*C*_3_); 77.83 (*C*_2_); 99.68 (*C*_1_); 100.10,109.76 ppm (2*C*(CH_3_)_2_); MS (ESI) *m/z*: 352.20 [M+Na]^+^, 368.02 [M+K]^+^.

*Monoisopropylidene derivative*: *R*_f_ = 0.26 (EtOAc/petroleum ether 3/2); ^1^H-NMR (acetone-*d*_6_ + D_2_O): *δ* = 1.27, 1.41 (2s, 6H, 2C*H*_3_); 3.45 (t, 2H, J = 5.0 Hz, C*H*_2_N_3_); 3.52 (m, 2H, *H*_4_ and *H*_5_); 3.62 (dd, 1H, *J* = 5.2 Hz, *J* = 11.6 Hz, *H*_6a_); 3.67 (m, 1H, C*H*_2_CH_2_N_3_); 3.80 (m, 1H, *H*_6b_); 3.93 (m, 1H, C*H*_2_CH_2_N_3_); 4.02 (m, 1H, *H*_3_); 4.09 (d, 1H, *J* = 5.6 Hz, *H*_2_); 5.03 (s, 1H, *H*_1_); ^13^C-NMR (acetone-*d*_6_ + D_2_O): *δ* = 20.34, 29.11 (2*C*H_3_); 51.95 (*C*H_2_N_3_); 62.97 (*C*_6_); 67.74 (*C*H_2_CH_2_N_3_); 70.41, 72.62 (*C*_4_ and *C*_5_); 77.30 (*C*_2_); 80.42 (*C*_3_); 98.60 (*C*_1_); 110.60 (*C*(CH_3_)_2_); MS (ESI) *m/z*: 312.12 [M+Na]^+^, 328.15 [M+K]^+^, 324.12 [M+Cl]^−^.

*2'-Azidoethyl-2,3-O-isopropylidene-α-d-mannopyranoside-4,6-cyclic sulfate* (**5**): Compound **4** (100 mg, 0.35 mmol, 1 eq.) and Et_3_N (144 µL, 1.04 mmol, 3 eq.) in CH_2_Cl_2_ (2 mL) were stirred for 5 min at 0 °C. Then SOCl_2_ (27 µL, 0.38 mmol, 1.1 eq.) was added dropwise to the mixture. After 10 min, the solution was filtered. Impurities were removed with water and the organic layer was washed with 1N HCl, dried (Na_2_SO_4_) and concentrated *in vacuo* to give a brown solid. The crude sulfite obtained was then reacted with NaIO_4_ (81 mg, 0.38 mmol, 1.1 eq.), water (0.5 mL) and RuCl_3_ (1.38.10^−3^ mmol, 0.004 eq.) in CH_2_Cl_2_/CH_3_CN 1:1 (2 mL). After 1h at RT, the solution was filtered before adding water. After extraction, the organic layer was dried (Na_2_SO_4_) and concentrated *in vacuo*. Filtration on silica gel and washes with CH_2_Cl_2_ gave a white solid (66%). *R*_f_ = 0.58 (EtOAc/petroleum ether 1:1); mp: 80–82 °C; ^1^H-NMR (acetone-*d*_6_): *δ* = 1.37, 1.52 (2s, 6H, 2C*H*_3_); 3.55 (m, 2H, C*H*_2_N_3_); 3.80 (m, 1H, C*H*_2_CH_2_N_3_); 4.29 (m, 1H, C*H*_2_CH_2_N_3_); 4.26 (td, 1H, *J* = 10.7 Hz, *J* = 5.5 Hz, *H*_5_); 4.36 (d, 1H, *J* = 6.0 Hz, *H*_2_); 4.43 (dd, 1H, *J* = 5.6 Hz, *J* = 8.0 Hz, *H*_3_); 4.6 (dd, 1H, *J* = 7.6 Hz, *J* = 10.8 Hz, *H*_4_); 4.63 (t, 1H, *J* = 10.8 Hz, *H*_6a_); 4.84 (dd, 1H, *J* = 5.6 Hz, *J* = 10.4 Hz, *H*_6b_); 5.28 ppm (s, 1H, *H*_1_); ^13^C-NMR (acetone-*d*_6_): *δ* = 27.16, 29.13 (2*C*H_3_); 52.06 (*C*H_2_N_3_); 60.35 (*C*_5_); 68.75 (*C*H_2_CH_2_N_3_); 74.34 (*C*_6_); 74.95 (*C*_3_); 77.88 (*C*_2_); 86.65 (*C*_4_); 99.70 (*C*_1_); 112.07 ppm (*C*(CH_3_)_2_); MS (ESI) *m/z*: 374.13 [M+Na]^+^, 386.08 [M+Cl]^−^.

3,6,9,12,15,18-Hexaoxatricos-22-en-1-ol (**6**): A mixture of 50% aqueous sodium hydroxide (1.93 mL, 24.18 mmol, 1.1 eq.) and hexa(ethylene glycol) (25 g, 88.55 mmol, 4.12 eq.) was stirred for 30 min at 100 °C, before adding 5-bromopent-1-ene (2.55 mL, 21.50 mmol, 1 eq.). After 15 min, the reaction mixture was cooled, diluted in CH_2_Cl_2_ and washed with water. The organic phase was dried (Na_2_SO_4_), filtered and concentrated *in vacuo*. Purification by chromatography on silica gel (EtOAc/petroleum ether 9:1 to EtOAc/MeOH 9:1) gave a yellow oil (99%). *R*_f_ = 0.14 (AcOEt/petroleum ether 5:5); 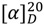 = + 54.9 (*c* = 1.00 in chloroform); ^1^H-NMR (CDCl_3_): *δ* = 1.68 (m, 2H, C*H*_2_CH_2_CH=CH_2_); 2.09 (m, 2H, C*H*_2_CH=CH_2_); 3.46 (t, 2H, *J* = 6.6 Hz, C*H*_2_CH_2_CH_2_CH=CH_2_); 3.56–3.73 (m, 24 h, 12C*H*_2_O); 4.99 (m, 2H, CH=C*H*_2_); 5.81 ppm (m, 2H, C*H=C*H_2_); ^13^C-NMR (CDCl_3_): *δ* = 28.66 (*C*H_2_CH_2_CH=CH_2_); 30.12 (*C*H_2_CH=CH_2_); 61.51–72.58 (13*C*H_2_O); 114.59 (CH=*C*H_2_); 138.18 ppm (*C*H=CH_2_); MS (ESI) *m/z*: 373.27 [M+Na]^+^, 389.20 [M+K]^+^.

*S-(23-Hydroxy-6,9,12,15,18,21-hexaoxatricos-1-yl)ethane-thioate* (**7**): A solution containing compound **6** (3.1 g, 8.85 mmol, 1 eq.), thiolacetic acid (3.17 mL, 44.28 mmol, 5 eq.) and AIBN (100 mg) in anhydrous THF (12 mL) was refluxed for 1 h under nitrogen. The mixture was diluted with EtOAc, washed with a saturated solution of NaHCO_3_. The organic layer was dried (Na_2_SO_4_), filtered and reduced *in vacuo*. Purification by chromatography on silica gel (EtOAc/petroleum ether 9:1 to EtOAc/MeOH 9:1) gave a yellow oil (71%). *R*_f_ = 0.27 (EtOAc/MeOH 9:1); ^1^H-NMR (CDCl_3_): *δ* = 1.40 (m, 2H, C*H*_2_(CH_2_)_2_S); 1.58 (m, 4 h, C*H*_2_(CH_2_)_3_S and C*H*_2_CH_2_S); 1.83 (s, 1H, OH); 2.32 (s, 3H, C*H*_3_); 2.86 (t, 2H, *J* = 7.2 Hz, C*H*_2_S); 3.44 (t, 2H, *J* = 6.6 Hz, C*H*_2_(CH_2_)_4_S); 3.56–3.73 ppm (m, 24 h, 12C*H*_2_O); ^13^C-NMR (CDCl_3_): *δ* = 25.25 (*C*H_2_(CH_2_)_2_S); 28.90 (*C*H_2_S); 28.99, 29.24 (*C*H_2_(CH_2_)_3_S and *C*H_2_CH_2_S); 30.52 (*C*H_3_); 61.55–72.43 (13*C*H_2_O); 195.84 ppm (*C*O); MS (ESI) *m/z*: 449.26 [M+Na]^+^, 461.17 [M+Cl]^−^.

*1-(Methoxytritylthio)-8,11,14,17,20,23-hexaoxa-2-thiapentacosan-25-ol* (**8**): Compound **7** (2.6 g, 6.1 mmol, 1 eq.) and a concentrated solution of HCl (3 mL) were stirred in EtOH (65 mL). After 20 h reaction at 60 °C, the mixture was neutralized with ammonia then reduced under pressure. The obtained solution was diluted with EtOAc, and the organic layer was quickly washed with water, dried (Na_2_SO_4_), and concentrated *in vacuo*. The crude product was directly put in reaction with MeOTrCl (2.83 g, 9.15 mmol, 1.5 eq.) in anhydrous THF (60 mL). After 24 h stirring at RT, the solution was concentrated in vacuo and purified by chromatography on silica gel (EtOAc/MeOH 9:1) to give a yellow oil (91%): *R*_f_ = 0.40 (EtOAc/MeOH 7:3); ^1^H-NMR (acetone-*d*_6_): *δ* = 1.31 (m, 2H, CH_2_(CH_2_)_2_S); 1.40 (m, 4 h, CH_2_(CH_2_)_3_S and CH_2_CH_2_S); 2.17 (t, 2H, *J* = 7.4 Hz, CH_2_S); 2.87 (s, 1H, OH); 3.35 (t, 2H, *J* = 6.4 Hz, CH_2_(CH_2_)_4_S); 3.47–3.63 (m, 24 h, 12CH_2_O); 3.79 (s, 3H, CH_3_); 6.86–7.42 ppm (m, 14 h, CH_Ar_); ^13^C-NMR (acetone-*d*_6_): *δ* = 27.37, 30.16 (*C*H_2_*C*H_2_*C*H_2_CH_2_S); 33.54 (*C*H_2_S); 56.50 (*C*H_3_); 62.94–72.33 (13*C*H_2_O); 74.48 (S*C*); 114.86–132.54 (14*C*H_Ar_); 138.81, 147.37 (3SC*C*_Ar_); 160.12 ppm (*C*OCH_3_); MS (ESI) *m/z*: 679.34 [M+Na]^+^.

*1-(Methoxytritylthio)-8,11,14,17,20,23-hexaoxa-2-thiahexacos-25-yne* (**9**): NaH (7.3 mg, 0.30 mmol, 2 eq.) and 2-bromopropyne (19 µL, 0.21 mmol, 1.4 eq.) were added to a solution containing compound **8** (100 mg, 0.15 mmol, 1 eq.) in anhydrous THF (3 mL) at 0 °C. After 18 h stirring at RT, the mixture was concentrated then purified by chromatography on silica gel (EtOAc/petroleum ether 8:2) to give a white oil (97%): *R*_f_ = 0.34 (EtOAc); ^1^H-NMR (CDCl_3_): *δ* = 1.28 (m, 2H, CH_2_(CH_2_)_2_S); 1.42 (m, 4 h, CH_2_(CH_2_)_3_S and CH_2_CH_2_S); 2.14 (t, 2H, *J* = 7.4 Hz, CH_2_S); 2.43 (t, 1H, *J* = 2.4 Hz, CH); 3.36 (t, 2H, *J* = 6.8 Hz, CH_2_(CH_2_)_4_S); 3.52–3.71 (m, 24 h, 12CH_2_O); 3.79 (s, 3H, CH_3_); 4.20 (d, 2H, *J* = 2.4 Hz, CH_2_CCH); 6.79–7.40 ppm (m, 14 h, CH_Ar_); ^13^C-NMR (CDCl_3_): *δ* = 25.59 (*C*H_2_(CH_2_)_2_S); 28.46, 29.19 (*C*H_2_(CH_2_)_3_S and *C*H_2_CH_2_S); 31.96 (*C*H_2_S); 55.20 (*C*H_3_); 58.39 (*C*H_2_CCH); 65.85 (*C*H); 69.10–71.17 (13*C*H_2_O); 74.51 (S*C*); 113.03–130.73 (14*C*H_Ar_); 137.12, 145.32 (3SC*C*_Ar_); 157.94 ppm (*C*OCH_3_); MS (ESI) *m/z*: 717.39 [M+Na]^+^.

*2-{4-[27-(4-Methoxyphenyl)-27,27-diphenyl-2,5,8,11,14,17,20-heptaoxa-26-thiaheptacos-1-yl]-2,3-dihydro-1H-1,2,3-triazol-1-yl}ethyl-6-deoxy-2,3-O-(1-methylethylidene)-4,6-cyclic sulfate-α-d-manno-pyranoside* (**10**): Compounds **5** (40 mg, 0.11 mmol, 1 eq.) and **9** (87 mg, 0.13 mmol, 1.1 eq.) were suspended in a mixture of *t*-BuOH/H_2_O 1:1 (4 mL). Cu(0) nanosize activated powder (4 mg, 0.06 mmol, 0.5 eq.) and NHEt_3_Cl (32 mg, 0.23 mmol, 2 eq.) were added, and the heterogeneous mixture was stirred vigorously for 20 h at RT. The reaction mixture was diluted in EtOAc then washed with water. The organic layer was dried, filtered and concentrated *in vacuo*. Purification by chromatography on silica gel (CH_2_Cl_2_/MeOH 99:1 to 9:1) gave a colourless oil (60%): *R*_f_ = 0.40 (CH_2_Cl_2_/MeOH 9:1); 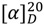 = + 36.0 (*c* = 1.00 in chloroform); ^1^H-NMR (CD_3_OD): *δ* = 1.36 (m, 6H, C*H*_2_C*H*_2_C*H*_2_CH_2_S); 1.34, 1.49 (2s, 6H, 2CC*H*_3_); 2.14 (t, 2H, *J* = 7.2 Hz, C*H*_2_S); 3.38 (t, 2H, *J* = 6.4 Hz, C*H*_2_(CH_2_)_4_S); 3.44–3.66 (m, 25 h, *H*_5_ and 12C*H*_2_O); 3.78 (s, 3H, OC*H*_3_); 3.97 (m, 1H, C*H*_2_CH_2_N); 4.15 (m, 1H, C*H*_2_CH_2_N); 4.27 (m, 3H, *H*_2_, *H*_3_ and *H*_6a_); 4.50 (m, 2H, *H*_6b_ and *H*_4_); 4.64 (m, 4 h, C*H*_2_N and C*H*_2_C=CH); 5.12 (s, 1H, *H*_1_); 6.81–7.39 (m, 14 h, C*H*_Ar_); 8.07 ppm (s, 1H, NC*H*); ^13^C-NMR (CD_3_OD): *δ* = 26.34, 28.20 (2C*C*H_3_); 26.71 (*C*H_2_(CH_2_)_2_S); 29.62 (*C*H_2_CH_2_S); 30.23 (*C*H_2_(CH_2_)_3_S); 33.03 (S*C*H_2_); 51.19 (*C*H_2_N); 55.79 (O*C*H_3_); 59.79 (*C*_5_); 65.14 (*C*H_2_C=CH); 67.42 (*C*H_2_CH_2_N); 70.95, 71.19, 71.49, 71.58, 72.03 (13*C*H_2_O); 73.53 (*C*_6_); 74.46, 77.23 (*C*_2_ and *C*_3_); 85.66 (*C*_4_); 98.96 (*C*_1_); 108.26, 111.66 (S*C* and *C*(CH_3_)_2_); 114.11, 127.66, 128.86, 130.73, 132.02 (14*C*H_Ar_); 126.04 (N*C*H); 138.40, 146.86 (3SC*C*_Ar_ and *C*=CH); 159.71 ppm (*C*OCH_3_); MS (ESI) *m/z*: 1068.62 [M+Na]^+^, 1080.77 [M+Cl]^−^.

*2-{4-[27-(4-Methoxyphenyl)-27,27-diphenyl-2,5,8,11,14,17,20-heptaoxa-26-thiaheptacos-1-yl]-2,3-dihydro-1H-1,2,3,-triazol-1-yl}ethyl-6-deoxy-2,3-O-(1-methylethylidene)-4-sodiumsulfate-6-azido-α-d-mannopyranoside* (**11**): The procedure described for compound **2** was applied to **10** to give compound **11** as a yellow oil (62%). *R*_f_ = 0.15 (CH_2_Cl_2_/MeOH 8.5:1.5); 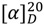 = +17.1 (*c* = 1.00 in chloroform); ^1^H-NMR (CD_3_OD): *δ* = 1.37 (m, 6H, C*H*_2_C*H*_2_C*H*_2_CH_2_S); 1.30, 1.45 (2s, 6H, 2CC*H*_3_); 2.17 (t, 2H, *J* = 7.2 Hz, C*H*_2_S); 3.37 (t, 2H, *J* = 6.6 Hz, C*H*_2_(CH_2_)_4_S); 3.42–3.67 (m, 27H, *H*_2_ and 13C*H*_2_O); 3.79 (s, 3H, OC*H*_3_); 3.96 (m, 1H, C*H*_2_CH_2_N); 4.12 (m, 1H, C*H*_2_CH_2_N); 4.07 (d, 1H, *J* = 6.0 Hz, *H*_5_); 4.25 (m, 1H, *H*_3_); 4.38 (t, 1H, *J* = 5.8 Hz, *H*_4_); 4.70 (m, 6H, *H*_6_, C*H*_2_N and C*H*_2_C=CH); 4.93 (s, 1H, *H*_1_); 6.87–7.41 (m, 14 h, C*H*_Ar_); 9.19 ppm (s, 1H, NC*H*); ^13^C-NMR (CD_3_OD): *δ* = 27.20, 28.67 (2C*C*H_3_); 27.33, 30.16, 30.87 (*C*H_2_*C*H_2_*C*H_2_CH_2_S); 33.55 (*C*H_2_S); 51.66 (*C*_6_ and *C*H_2_(CH_2_)_4_S); 54.55 (*C*H_2_N); 56.53 (O*C*H_3_); 65.58 (*C*H_2_C=CH); 67.89 (*C*H_2_CH_2_N); 70.39–72.33 (12*C*H_2_O); 72.13 (*C*_2_); 73.88 (*C*_3_); 76.31 (*C*_5_); 77.37 (*C*_4_); 99.59 (*C*_1_); 110.79 (S*C*); 114.87–132.54 (14*C*H_Ar_); 126.23 (N*C*H); 138.80–160.13 (3SC*C*_Ar_ and *C*=CH); 158.40 ppm (*C*OCH_3_); MS(ESI) *m/z*:1134.58 [M+Na]^+^.

*2-{4-[27-(4-Methoxyphenyl)-27,27-diphenyl-2,5,8,11,14,17,20-heptaoxa-26-thiaheptacos-1-yl]-2,3-dihydro-1H-1,2,3,-triazol-1-yl}ethyl-6-deoxy-6-azido-α-d-mannopyranoside* (**12**): Firstly, compound **11** (200 mg, 0.18 mmol, 1 eq.) and CAN (50 mg, 0.09 mmol, 0.5 eq.) were added to a mixture of CH_3_CN/H_2_O 95:5 (4 mL). After 4 h stirring at 60 °C, the solution was diluted in CH_2_Cl_2_, washed several times with water and the aqueous layer was lyophilized. Purification by chromatography on silica gel (CH_2_Cl_2_/MeOH 9:1 to CH_2_Cl_2_/MeOH 8:2) gave the product as a colourless oil (72%). Secondly, this intermediate was dissolved in a mixture of MeOH/THF 1:1 (6 mL) before adding Amberlyst H^+^ resins. After 24 h at RT, the resins were filtered, and the solution was neutralized with a saturated solution of NaHCO_3_. Organic solvents were evaporated and water lyophilized. The crude product was dissolved in methanol and the insoluble NaHCO_3_ was filtered. Purification by chromatography on silica gel (CH_2_Cl_2_/MeOH 9:1) gave the product as a colourless oil (53%): *R*_f_ = 0.25 (CH_2_Cl_2_/MeOH 9:1); 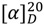 = −2.1 (*c* = 1.00 in chloroform); ^1^H-NMR (CD_3_OD): *δ* = 1.47 (m, 2H, C*H*_2_(CH_2_)_2_S); 1.60 (m, 2H, C*H*_2_(CH_2_)_3_S); 1.71 (m, 2H, C*H*_2_CH_2_S); 2.70 (t, 2H, *J* = 7.2 Hz, C*H*_2_S); 3.48 (t, 2H, *J* = 6.2 Hz, C*H*_2_(CH_2_)_4_S); 3.19–3.78 (m, 30H, *H*_2-6_ and 12C*H*_2_O); 3.88 (m, 1H, C*H*_2_CH_2_N); 4.13 (m, 1H, C*H*_2_CH_2_N); 4.63 (m, 4 h, C*H*_2_N and C*H*_2_C=CH); 4.72 (s, 1H, *H*_1_); 8.03 ppm (s, 1H, NC*H*); ^13^C-NMR (CD_3_OD): *δ* = 26.13 (*C*H_2_(CH_2_)_3_S); 30.07 (*C*H_2_CH_2_S); 30.36 (*C*H_2_(CH_2_)_3_S); 39.66 (*C*H_2_S); 51.34 (*C*H_2_N); 62.85 (*C*_6_); 65.05 (*C*H_2_C=CH); 66.79 (*C*H_2_CH_2_N); 68.38, 70.81, 71.24, 71.59, 71.93, 72.15, 72.51, 75.01 (*C*_2-5_ and 13*C*H_2_O); 101.70 (*C*_1_); 132.57 (N*C*H); 161.04 ppm (*C*H=C); MS (ESI) *m/z*: 798.62 [M+Na]^+^.

*2-{4-[27-(4-Methoxyphenyl)-27,27-diphenyl-2,5,8,11,14,17,20-heptaoxa-26-thiaheptacos-1-yl]-2,3-dihydro-1H-1,2,3,-triazol-1-yl}ethyl-6-deoxy-2,3-O-(1-methylethylidene)-4-sodium sulfate-6-cyano-α-d-mannopyranoside* (**13**): Sodium cyanide (15 mg, 0.31 mmol, 2 eq.) was added to a suspension of compound **10** (160 mg, 0.15 mmol, 1 eq.) in DMF (1.5 mL). After 4 h stirring at RT, the mixture was poured into brine and extracted with CH_2_Cl_2_. The organic layers were dried (Na_2_SO_4_) and concentrated in vacuo. The residue was purified by chromatography on silica gel (CH_2_Cl_2_/MeOH 9:1) to give the appropriated intermediate as a colourless oil (65%): *R*_f_ = 0.24 (CH_2_Cl_2_/MeOH 9:1); ^1^H-NMR (CD_3_OD): *δ* = 1.31, 1.50 (2s, 6H, 2CC*H*_3_); 1.38 (m, 6H, C*H*_2_C*H*_2_C*H*_2_CH_2_S); 2.15 (t, 2H, *J* = 7.2 Hz, C*H*_2_S); 2.70 (dd, 1H, *J* = 8.8 Hz, *J* = 17.2 Hz, *H*_6a_); 3.02 (dd, 1H, *J* = 3.0 Hz, *J* = 17.0 Hz, *H*_6b_); 3.38 (t, 2H, *J* = 6.4 Hz, C*H*_2_(CH_2_)_4_S); 3.48–3.66 (m, 26H, *H*_4,5_ and 12C*H*_2_O); 3.78 (s, 3H, OC*H*_3_); 3.93 (m, 1H, C*H*_2_CH_2_N); 4.10 (d, 1H, *J* = 4.8 Hz, *H*_2_); 4.19 (m, 2H, C*H*_2_CH_2_N and *H*_3_); 4.65 (m, 4 h, C*H*_2_N and C*H*_2_C=CH); 5.00 (s, 1H, *H*_1_); 6.82–7.39 (m, 14 h, C*H*_Ar_); 8.04 ppm (s, 1H, NC*H*); ^13^C-NMR (100.62 MHz, CD_3_OD): *δ* = 21.83 (*C*_6_); 26.60, 28.05 (2C*C*H_3_); 26.69 (*C*H_2_(CH_2_)_2_S); 29.62 (*C*H_2_CH_2_S); 30.17 (*C*H_2_(CH_2_)_3_S); 33.04 (*C*H_2_S); 51.17 (*C*H_2_N); 55.81 (O*C*H_3_); 64.87 (*C*H_2_C=CH); 66.66, 76.70 (*C*_4_ and *C*_5_); 67.02 (*C*H_2_CH_2_N); 70.47–72.02 (13*C*H_2_O); 77.54 (*C*_2_); 77.92 (*C*_3_); 98.52 (*C*_1_); 110.98 (S*C* and *C*(CH_3_)_2_); 118.96 (CH_2_*C*N); 114.12–132.01 (14*C*H_Ar_); 125.87 (N*C*H); 138.38, 146.00, 146.84 (3SC*C*_Ar_ and *C*=CH); 159.71 ppm (*C*OCH_3_); MS (ESI) *m/z*: 1117.77 [M+Na]^+^,1071.63 [M-Na]^−^.

*2-{4-[27-(4-Methoxyphenyl)-27,27-diphenyl-2,5,8,11,14,17,20-heptaoxa-26-thiaheptacos-1-yl]-2,3-dihydro-1H-1,2,3,-triazol-1-yl}ethyl-6-deoxy-α-d-heptomannopyranouronic acid* (**14**): Firstly, NaOH (60 mg, 1.46 mmol, 8 eq.) was added to a solution of compound **13** (200 mg, 0.18 mmol, 1 eq.) and H_2_O_2_ at 30% (1.5 mL). 1.5 mL of H_2_O_2_ at 30% and 60 mg of NaOH were added to the mixture after 12 h stirring at RT and again after 24 h stirring at RT. After 48 h, the solution was neutralized with Amberlite IRC-50 (H^+^) resin, filtered, and concentrated *in vacuo*. The crude product was purified by chromatography on silica gel (CH_2_Cl_2_/MeOH 9:1 to NH_4_OH/iPrOH 1:1) to give a yellow oil (52%): *R*_f_ = 0.15 (AcOEt/MeOH 5:5); 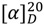 = −9.0 (*c* = 1.00 in chloroform); ^1^H-NMR (D_2_O): *δ* = 1.35, 1.52 (2s, 6H, 2CC*H*_3_); 1.44 (m, 2H, C*H*_2_(CH_2_)_2_S); 1.60 (m, 2H, C*H*_2_(CH_2_)_3_S); 1.73 (m, 2H, C*H*_2_CH_2_S); 2.29 (dd, 1H, *J* = 10.6 Hz, *J* = 15.0Hz, *H*_6a_); 2.80 (dd, 1H, *J* = 2.0 Hz, *J* = 15.2 Hz, *H*_6b_); 2.89 (t, 2H, *J* = 8.0 Hz, C*H*_2_S); 3.53 (t, 2H, *J* = 6.6 Hz, C*H*_2_(CH_2_)_4_S); 3.50–3.69 (m, 25 h, *H*_5_ and 12C*H*_2_O); 3.75 (s, 3H, OC*H*_3_); 3.88 (m, 1H, C*H*_2_CH_2_N); 4.17 (m, 2H, C*H*_2_CH_2_N and *H*_4_); 4.19 (d, 1H, *J* = 5.6 Hz, *H*_2_); 4.30 (m, 1H, *H*_3_); 4.68 (m, 4 h, C*H*_2_N and C*H*_2_C=CH); 4.95 (s, 1H, *H*_1_); 6.80–7.37 (m, 14 h, C*H*_Ar_); 8.12 ppm (s, 1H, NC*H*); ^13^C-NMR (D_2_O): *δ* = 23.80 (*C*H_2_CH_2_S); 24.24 (*C*H_2_(CH_2_)_2_S); 25.45, 26.80 (2C*C*H_3_); 28.06 (*C*H_2_(CH_2_)_3_S); 38.92 (*C*_6_); 49.99 (*C*H_2_N); 50.89 (*C*H_2_S); 55.86 (O*C*H_3_); 62.95 (*C*H_2_C=CH); 65.47 (*C*H_2_CH_2_N); 66.41 (*C*_5_); 66.54, 68.69, 69.07, 69.40, 69.53, 70.70 (13*C*H_2_O); 75.21 (*C*_2_); 76.00 (*C*_3_); 78.30 (*C*_4_); 95.99 (*C*_1_); 110.39 (S*C* and *C*(CH_3_)_2_); 114.14–132.02 (14*C*H_Ar_); 125.55 (N*C*H); 143.85 (*C*=CH); 138.38, 146.00, 146.84 (3SC*C*_Ar_); 159.71 (*C*OCH_3_);177.75 (*C*O_2_H); MS (ESI) *m/z*: 1037.34 [M+Na]^+^. 

Next, the procedure described for compound **12** was applied to the preceeding intermediate to give compound **14** as a colourless oil (80%). *R*_f_ = 0.18 (EtOAc/MeOH 5:5); 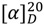 = +10.2 (*c* = 1.00 in chloroform); ^1^H-NMR (CD_3_OD): *δ* = 1.49 (m, 2H, C*H*_2_(CH_2_)_2_S); 1.61 (m, 2H, C*H*_2_(CH_2_)_3_S); 1.80 (m, 2H, C*H*_2_CH_2_S); 2.41 (dd, 1H, *J* = 10.2 Hz, *J* = 16.2 Hz, *H*_6a_); 2.84 (m, 3H, C*H*_2_S and *H*_6b_); 3.49 (t, 2H, *J* = 6.4 Hz, C*H*_2_(CH_2_)_4_S); 3.40–3.79 (m, 28 h, *H*_2-5_ and 12 C*H*_2_O); 3.92 (m, 1H, C*H*_2_CH_2_N); 4.22 (m, 1H, C*H*_2_CH_2_N); 4.71 (d, 1H, *J* = 1.2 Hz, *H*_1_); 4.87 (m, 2H, C*H*_2_N); 4.92 (m, 2H, C*H*_2_C=CH); 8.65 (s, 1H, NC*H*); ^13^C-NMR (D_2_O): *δ* = 23.79 (*C*H_2_CH_2_S); 24.23 (*C*H_2_(CH_2_)_2_S); 28.06 (*C*H_2_(CH_2_)_3_S); 36.51 (*C*_6_); 50.67 (*C*H_2_N); 50.90 (*C*H_2_S); 62.55 (*C*H_2_C=CH); 65.29 (*C*H_2_N); 67.88, 69.08, 69.54, 70.70 (13*C*H_2_O); 52.32, 69.36, 69.82, 70.16 (*C*_2-5_); 99.48 (*C*_1_); 109.39 (*C*=CH); 146.74 (N*C*H); 175.27 ppm (*C*O_2_H); MS (ESI) *m/z*: 765.86 [M-3H+3Na]^+^.

*Methyl-2,3-O-isopropylidene-4,6-cyclic sulfate-α-d-mannopyranoside* (**15**): Firstly, the procedure described for compound **4** was applied to methyl α-d-mannopyranoside to give the appropriate intermediate as a white solid (63%): *R*_f_ = 0.53 (EtOAc); mp: 103–105 °C; ^1^H-NMR (acetone-*d*_6_): *δ* = 1.28, 1.42 (2s, 6H,2CC*H*_3_); 3.35 (s, 3H, OC*H*_3_); 3.46 (ddd, 1H, *J* = 2.6 Hz, *J* = 5.6 Hz, *J* = 10.2 Hz, *H*_5_); 3.52 (dd, 1H,*J* = 6.9 Hz, *J* = 10.2 Hz, *H*_4_); 3.65 (dd, 1H, *J* = 5.8 Hz, *J* = 12.0 Hz, *H*_6a_); 3.81 (dd, 1H, *J* = 2.6 Hz, *J* = 11.9Hz, *H*_6b_); 4.01 (dd, 1H, *J* = 5.7 Hz, *J* = 6.9 Hz, *H*_3_); 4.06 (dd, 1H, *J* = 0.8 Hz, *J* = 5.7 Hz, *H*_2_); 5.01 ppm (s, 1H,*H*_1_); ^13^C-NMR (acetone-*d*_6_+D_2_O): *δ* = 26.50, 28.31 (2C*C*H_3_), 55.57 (O*C*H_3_), 62.79 (*C*_6_), 69.74, 70.02 (*C*_4_,*C*_5_), 75.97 (*C*_2_), 78.83 (*C*_3_), 98.80 (*C*_1_), 110.07 ppm (*C*(CH_3_)_2_); MS (ESI): *m*/*z* [*M*+Na]^+^ calcd. for C_10_H_18_O_6_ Na: 257.10, found: 257.21.

Next, the procedure described for compound **5** was applied to the preceeding intermediate to give compound **15** as a white solid (85%). mp: 80–82 °C; *R*_f_ = 0.48 (EtOAc/petroleum ether 3:7); ^1^H-NMR (acetone-*d*_6_): *δ* = 1.38, 1.53 (2s, 6H, 2CC*H*_3_); 3.46 (s, 3H, OC*H*_3_); 4.17 (td, 1H, *J* = 5.5 Hz, *J* = 10.6 Hz, *H*_5_); 4.32 (dd, 1H, *J* = 0.4 Hz, *J* = 5.6 Hz, *H*_2_); 4.42 (dd, 1H, *J* = 5.6 Hz, *J* = 7.7 Hz, *H*_3_); 4.59 (dd, 1H, *J* = 7.8 Hz, *J* = 10.4 Hz, *H*_4_); 4.64 (t, 1H, *J* = 10.7 Hz, *H*_6a_); 4.87 (dd, 1H, *J* = 5.5 Hz, *J* = 10.5 Hz, *H*_6b_); 5.01 (d, 1H, *J* = 0.5 Hz, *H*_1_); ^13^C-NMR (CDCl_3_): *δ* = 26.44, 28.45 (2C*C*H_3_); 56.14 (O*C*H_3_); 58.94 (*C*_5_); 72.32 (*C*_6_); 76.37 (*C*_2_); 73.61 (*C*_3_); 84.67 (*C*_4_); 99.42 (*C*_1_); 111.08 ppm (*C*(CH_3_)_2_); MS (ESI) *m/z*: 297.37 [M+H]^+^, 319.32 [M+Na]^+^.

*Methyl-6-deoxy-6-azido-2,3,4-tri-O-acetyl-α-d-mannopyranoside* (**16**): First, the procedures described for compounds **2** and **4** were applied to **15** to give **16** as a white solid: *R*_f_ = 0.50 (CH_2_Cl_2_/MeOH 9:1); 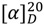 = +54.8 (*c* = 1.00 in methanol); ^1^H-NMR (D_2_O): *δ* = 3.40 (s, 3H, OC*H*_3_); 3.54 (dd, 1H, *J* = 6.2 Hz, *J* = 13.3 Hz, *H*_6a_); 3.60–3.73 (m, 4 h, *H*_6b_, *H*_5_, *H*_4_ and *H*_3_); 3.91 (dd, 1H, *J* = 3.3 Hz, *J* = 1.7 Hz, *H*_2_); 4.73 (d, 1H, *J* = 1.6 Hz, *H*_1_); ^13^C-NMR (D_2_O): *δ* = 51.4 (*C*_6_); 55.2 (O*C*H_3_); 67.8 (*C*_5_); 70.2 (*C*_2_); 70.7 (*C*_3_); 71.6 (*C*_4_); 101.4 ppm (*C*_1_); MS(ESI) *m/z*:242.31 [M+Na]^+^, 218.14 [M-H]^−^.

Secondly, Ac_2_O (1.72 mL, 18.26 mmol, 5 eq.) and DMAP (134 mg, 1.10 mmol, 0.3 eq.) were added to a solution of pyridine (15 mL) and methyl 6-azido-6-deoxy-α-d-mannopyranoside (800 mg, 3.65 mmol, 1 eq.). After 4 h stirring, the mixture was diluted in ethyl acetate and washed with a solution of HCl 2N (until pH = 1), a solution of NaHCO_3_ 5%, water (until pH = 7) and with a saturated solution of NaCl. The organic layer was dried (Na_2_SO_4_), filtered and concentrated *in vacuo*. The residue was purified by chromatography on silica gel (EtOAc/petroleum ether 2:3) to give a yellow powder (97%). *R*_f_ = 0.62 (EtOAc/petroleum ether 1:1); mp: 98–100 °C (lit. 99–100 °C); 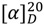 = +65.7 (*c* = 1.00 in chloroform); ^1^H-NMR (CDCl_3_): *δ* = 1.97, 2.05, 2.13 (3s, 9H, COC*H*_3_); 3.16 (dd, 1H, *J* = 8.8 Hz, *J* = 10.8 Hz, *H*_6a_); 3.29 (dd, 1H, *J* = 2.6 Hz, *J* = 11.0 Hz, *H*_6b_); 3.46 (s, 3H, OC*H*_3_); 3.78 (td, 1H, *J* = 2.4 Hz, *J* = 9.2 Hz, *H*_5_); 4.71 (s, 1H, *J* = 1.2 Hz, *H*_1_); 5.09 (t, 1H, *J* = 9.8 Hz, *H*_4_); 5.20 (m, 1H, *H*_2_); 5.29 ppm (dd, 1H, *J* = 3.6 Hz, *J* = 10.0 Hz, *H*_3_); ^13^C-NMR (CDCl_3_): *δ* = 3.85 (*C*_6_); 20.60, 20.73, 20.80 (3CO*C*H_3_); 55.49 (O*C*H_3_); 68.60 (*C*_3_); 69.52 (*C*_2_); 69.90 (*C*_4_); 70.07 (*C*_5_); 98.44 (*C*_1_); 169.77, 169.80, 169.95 ppm (3*C*=O); MS (ESI) *m/z*: 368.24 [M+Na]^+^.

(*6-Deoxy-6-azido-1,2,3,4-tetra-O-acetyl-α-d-mannopyranose* **17**): Compound **16** (500 mg, 1.45 mmol, 1 eq.) dissolved in acetic anhydride (10 mL) was added dropwise to a solution of Ac_2_O/AcOH/H_2_SO_4_ 5:4:1 (12.5 mL) at 0 °C. After 4 h at RT, the mixture was diluted with EtOAc then ice was added slowly. The obtained organic layer was washed with a solution of NaHCO_3_ 5% then water, dried (Na_2_SO_4_), filtered and concentrated *in vacuo*. The beige oil was used without purification (83%): *R*_f_ = 0.83 (CH_2_Cl_2_/MeOH 9:1); 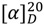 = −42.7 (*c* = 1.01 in chloroform); ^1^H-NMR (CDCl_3_): *δ* = 1.98, 2.03, 2.14, 2.15 (4s, 12H, 4C*H*_3_); 3.28 (dd, 1H, *J* = 5.6 Hz, *J* = 13.6 Hz, *H*_6a_); 3.37 (dd, 1H, *J* = 2.4 Hz, *J* = 13.2 Hz, *H*_6b_); 3.97 (m, 1H, *H*_5_); 5.22 (s, 1H, *H*_2_); 5.31 (m, 2H, *H*_3_ and *H*_4_); 6.06 ppm (d, 1H, *J* = 1.6 Hz, *H*_1_); ^13^C-NMR (CDCl_3_): *δ* = 20.53, 20.56, 20.63, 20.72 (4*C*H_3_); 50.55 (*C*_6_); 66.30, 68.43 (*C*_3_ and *C*_4_); 68.14 (*C*_2_); 71.70 (*C*_5_); 90.19 (*C*_1_); 167.96, 169.49, 169.68, 169.92 ppm (4*C*=O); MS (ESI) *m/z*: 396.22 [M+Na]^+^, 408.35 [M-Cl]^−^.

*4-Bromobut-2-en-1-yl-6-deoxy-6-azido-2,3,4-tri-O-acetyl-α-d-mannopyranoside* (**18**): The procedure described for compound **1** was applied to **17** and 4-bromo-but-2-en-1-ol to give compound **18** as a beige oil (78%): *R*_f_ = 0.59 (EtOAc/petroleum ether 1:1); ^1^H-NMR (CDCl_3_): *δ* = 1.97, 2.03, 2.13 (3s, 9H, 3C*H*_3_); 3.25 (dd, 1H, *J* = 5.6 Hz, *J* = 13.5 Hz, *H*_6a_); 3.32 (dd, 1H, *J* = 2.4 Hz, *J* = 13.5 Hz, *H*_6b_); 3.99 (m, 1H, *H*_5_); 4.01 (d, 2H, *J* = 8.4 Hz, C*H*_2_Br); 4.68 (m, 2H, C*H*_2_O); 4.96 (d, 1H, *J* = 1.6 Hz, *H*_1_); 5.45 (m, 2H, *H*_3_ and *H*_4_); 5.62 (s, 1H, *H*_2_); 5.71 (m, 1H, C*H*CH_2_O); 5.93 ppm (m, 1H, C*H*CH_2_Br); ^13^C-NMR (CDCl_3_): *δ* = 20.51, 20.54, 20.62 (3*C*H_3_); 25.66 (*C*H_2_Br); 50.54 (*C*_6_); 59.12 (*C*H_2_O); 66.33, 68.46 (*C*_3_ and *C*_4_); 68.34 (*C*_2_); 71.71 (*C*_5_); 90.23 (*C*_1_); 128.10 (*C*HCH_2_O); 129.75 (*C*HCH_2_Br); 167.97, 169.52, 169.70 ppm (3*C*=O); MS (ESI) *m/z*: 487.45 [M+Na]^+^.

*Methyl 6-deoxy-2,3-O-(1-methylethylidene)-4-O-sodiumsulfate-α-d-heptomanno-pyranosiduronic acid* (**19**): Firstly, the procedure described for compound **13** was applied to **15** to give the appropriate intermediate as a yellow solid (quantitative): *R*_f_ = 0.49 (CH_2_Cl_2_/MeOH 8.5:1.5); 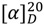 = + 37.7 (*c* = 1.00 in chloroform); ^1^H-NMR (acetone-*d*_6_): *δ* = 1.24, 1.41 (2s, 6H, 2CC*H*_3_); 2.76 (dd, 1H, *J* = 9.3 Hz, *J* = 17.3 Hz, *H*_6a_); 3.18 (dd, 1H, *J* = 2.8 Hz, *J* = 17.3 Hz, *H*_6b_); 3.46 (s, 3H, OC*H*_3_); 3.86 (td, 1H, *J* = 9.6 Hz, *J* = 2.8 Hz, *H*_5_); 4.15 (d, 1H, *J* = 7.4 Hz, *H*_2_); 4.21 (dd, 1H, *J* = 9.9 Hz, *J =* 7.0 Hz, *H*_4_); 4.44 (m, 1H, *H*_3_); 4.93 ppm (s, 1H, *H*_1_); ^13^C-NMR (acetone-*d*_6_): *δ* = 20.60 (*C*_6_); 25.5, 27.10 (2C*C*H_3_); 54.5 (O*C*H_3_); 64.90 (*C*_5_); 75.62 (*C*_2_); 76.34 (*C*_4_); 76.90 (*C*_3_); 98.17 (*C*_1_); 109.88 (*C*(CH_3_)_2_); 118.13 ppm (*C*N); MS (ESI) *m/z*: 384.23 [M+Na]^+^, 322.42 [M-Na]^−^.

Secondly, the procedure described for compound **14** was applied to the precedent intermediate to give compound **19** as a colourless oil (quantitative): *R*_f_ = 0.61 (EtOAc/MeOH 1:1); 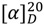 = +17.23 (*c* = 1.00 in chloroform); ^1^H-NMR (CD_3_OD): *δ* = 1.33, 1.53 (2s, 6H, 2CC*H*_3_); 2.40 (dd, 1H, *J* = 9.8 Hz, *J* = 15.8 Hz, *H*_6a_); 3.09 (dd, 1H, *J* = 2.2 Hz, *J* = 16.2 Hz, *H*_6b_); 3.41 (s, 3H, OC*H*_3_); 4.09 (m, 2H, *H*_2_ and *H*_5_); 4.21 (m, 2H, *H*_3_ and *H*_4_); 4.81 ppm (s, 1H, *H*_1_); ^13^C-NMR (CD_3_OD): *δ* = 26.57, 28.12 (2C*C*H_3_); 38.32 (*C*_6_); 55.76 (O*C*H_3_); 66.91 (*C*_2_); 77.19, 78.07, 79.05 (*C*_3_, *C*_4_ and *C*_5_); 99.42 (*C*_1_); 110.75 (*C*(CH_3_)_2_); 175.20 ppm (*C*O_2_H); MS (ESI) *m/z*: 387.99 [M+Na]^+^, 363.12 [M-H]^−^.

*Methyl 6-deoxy-2,3-4-tri-O-acetyl-α-d-heptomannopyranosiduronic acid* (**20**): Firstly, the procedure described for compound **4** was applied to **19** to give the appropriate intermediate as a colourless oil (78%): *R*_f_ = 0.25 (*i*-PrOH/NH_4_OH 8.5:1.5); ^1^H-NMR (D_2_O): *δ* = 2.86 (dd, 1H, *J* = 7.4 Hz, *J* = 17.3 Hz, *H*_6a_); 3.04 (dd, 1H, *J* = 3.6 Hz, *J* = 17.3 Hz, *H*_6b_); 3.44 (s, 3H, OC*H*_3_); 3.60 (t, 1H, *J* = 9.7 Hz, *H*_4_); 3.76 (dd, 1H, *J* = 9.6 Hz, *J* = 3.4 Hz, *H*_3_); 3.84 (m, 1H, *H*_5_); 3.96 (dd, 1H, *J* = 3.4 Hz, *J* = 1.7 Hz, *H*_2_); 4.78 ppm (d, 1H, *J* = 1.5 Hz, *H*_1_); ^13^C-NMR (D_2_O): *δ* = 51.44 (*C*_6_); 55.20 (O*C*H_3_); 67.89 (*C*_5_); 70.27 (*C*_2_); 70.76 (*C*_3_); 71.60 (*C*_4_); 101.42 (*C*_1_); 176.01 ppm (*C*O_2_H); MS (ESI) *m/z*: 245.56 [M+Na]^+^, 221.03 [M-H]^−^.

Secondly, the procedure described for compound **16** was applied to the preceeding intermediate to give compound **20** as a white powder (92%): *R*_f_ = 0.71 (EtOAc/petroleum ether 1:1); ^1^H-NMR (CDCl_3_): *δ* = 1.98, 2.03, 2.10 (3s, 9H, 3COC*H*_3_); 3.40 (s, 3H, OC*H*_3_); 3.96 (m, 1H, *H*_5_); 4.11 (dd, 1H, *J* = 2.4 Hz, *J* = 12.4 Hz, *H*_6a_); 4.28 (dd, 1H, *J* = 5.4 Hz, *J* = 12.2 Hz, *H*_6b_); 4.71 (d, 1H, *J* = 1.6 Hz, *H*_1_); 5.23 (m, 1H, *H*_2_); 5.27 (t, 1H, *J* = 9.8 Hz, *H*_4_); 5.32 ppm (dd, 1H, *J* = 3.2 Hz, *J* = 10.0 Hz, *H*_3_); ^13^C-NMR (CDCl_3_): *δ* = 20.67, 20.72, 20.87 (3CO*C*H_3_); 55.28 (O*C*H_3_); 62.46 (*C*_6_); 66.08 (*C*_4_); 68.32 (*C*_5_); 69.00 (*C*_3_); 69.45 (*C*_2_); 98.54 (*C*_1_); 169.88, 170.04, 170.66 (3*C*=O); 175.89 ppm (*C*O_2_H); MS (ESI) *m/z*: 371.59 [M+Na]^+^.

*6-Deoxy-1-2,3-4-tetra-O-acetyl-α-d-heptomannopyranosiduronic acid* (**21**): The procedure described for compound **17** was applied to **20** to give compound **21** as a beige oil (83%): *R*_f_ = 0.48 (EtOAc/petroleum ether 1:1); ^1^H-NMR (CDCl_3_): *δ* = 1.95, 2.06, 2.12, 2.15 (4s, 12H, 4C*H*_3_); 3.91 (m, 1H, *H*_5_); 4.17 (dd, 1H, *J* = 2.7 Hz, *J* = 12.6 Hz, *H*_6a_); 4.32 (dd, 1H, *J* = 5.4 Hz, *J* = 12.2 Hz, *H*_6b_); 5.30 (t, 1H, *J* = 9.9 Hz, *H*_4_); 5.34 (m, 1H, *H*_2_); 5.35 (dd, 1H, *J* = 3.2 Hz, *J* = 10.0 Hz, *H*_3_); 5.98 ppm (s, 1H, *H*_1_); ^13^C-NMR (CDCl_3_): *δ* = 20.67, 20.72, 20.87, 20.90 (4*C*H_3_); 62.44 (*C*_6_); 66.12 (*C*_4_); 68.36 (*C*_5_); 68.95 (*C*_3_); 69.43 (*C*_2_); 90.53 (*C*_1_); 169.72, 169.88, 170.04, 170.66 (4*C*=O); 176.08 ppm (*C*O_2_H); MS (ESI) *m/z*: 399.89 [M+Na]^+^.

*4-Bromobut-2-en-1-yl-6-deoxy-2,3,4-tri-O-acetyl-α-d-heptomannopyranosiduronic acid* (**22**): The procedure described for compound **18** was applied to **21** and 4-bromo-but-2-en-1-ol to give compound **22** as a beige oil (78%): *R*_f_ = 0.23 (EtOAc/petroleum ether 1:1); ^1^H-NMR (CDCl_3_): *δ* = 1.95, 2.06, 2.12 (3s, 9H, 3C*H*_3_); 3.91 (m, 1H, *H*_5_); 4.08 (d, 2H, *J* = 8.6 Hz, C*H*_2_Br); 4.17 (dd, 1H, *J* = 2.7 Hz, *J* = 12.6 Hz, *H*_6a_); 4.32 (dd, 1H, *J* = 5.4 Hz, *J* = 12.2 Hz, *H*_6b_); 4.70 (m, 2H, C*H*_2_O); 5.30 (t, 1H, *J* = 9.9 Hz, *H*_4_); 5.34 (m, 1H, *H*_2_); 5.35 (dd, 1H, *J* = 3.2 Hz, *J* = 10.0 Hz, *H*_3_); 5.77 (m, 1H, C*H*CH_2_O); 5.90 (m, 1H, C*H*CH_2_Br); 5.98 ppm (s, 1H, *H*_1_); ^13^C-NMR (CDCl_3_): *δ* = 20.65, 20.70, 20.80 (3*C*H_3_); 25.62 (*C*H_2_Br); 59.26 (*C*H_2_O); 62.48 (*C*_6_); 66.18 (*C*_4_); 68.39 (*C*_5_); 69.00 (*C*_3_); 69.40 (*C*_2_); 90.51 (*C*_1_); 128.15 (*C*HCH_2_O); 129.77 (*C*HCH_2_Br); 169.67, 169.83, 170.06 (3*C*=O); 176.09 ppm (*C*O_2_H); MS (ESI) *m/z*: 490.05 [M+Na]^+^.

*(2E)-1-Bromo-3,7-dimethylocta-2,6-diene* (**23**): PBr_3_ (1.1 mL, 9.51 mmol, 0.33 eq.) was added dropwise at 0 °C to geraniol (5 mL, 28.83 mmol, 1 eq.) dissolved in CH_2_Cl_2_ (50 mL). After 1 h at 0 °C, the mixture was diluted in CH_2_Cl_2_ and ice-cubes were added.The obtained organic layer was washed with a solution of NaHCO_3_ 5% and water, dried (Na_2_SO_4_), filtered and concentrated *in vacuo*. Purification by chromatography on silica gel (EtOAc/petroleum ether 1:1) gave a yellow oil (93%): *R*_f_ = 0.80 (EtOAc/petroleum ether 1:1); ^1^H-NMR (CDCl_3_): *δ* = 1.59, 1.66, 1.67 (2s, 9H, 3C*H*_3_); 2.01,2.09 (2m, 4 h, C*H*_2_C*H*_2_); 4.13 (d, 2H, *J* = 7.2 Hz, C*H*_2_Br); 5.08 (m, 1H, C*H*C=C(CH_3_)_2_); 5.40 ppm (m, 1H, C*H*CH_2_Br); ^13^C-NMR (CDCl_3_): *δ* = 16.20, 17.62,25.62 (3*C*H_3_); 26.31,39.48 (*C*H_2_*C*H_2_); 59.29 (*C*H_2_Br); 123.28 (*C*HCH_2_Br); 123.83 (*C*HC=C(CH_3_)_2_); 131.68, 139.61 ppm (2*C*=CH); MS (ESI) *m/z*: 241.67 [M+Na]^+^.

*{[(2E)-3,7-Dimethylocta-2,6-dien-1-yl]sulfonyl}benzene* (**24**): Compound **23** (6 g, 27.52 mmol, 1 eq.) and NaSO_2_Ph (9 g, 55.05 mmol, 2eq.) were stirred in DMF (12 mL) for 2 h at RT. Then the solution was concentrated and the crude product was purified by chromatography on silica gel (EtOAc/petroleum ether 1:1) to give a yellow oil (93%): *R*_f_ = 0.45 (EtOAc /petroleum ether 3:7); ^1^H-NMR (CDCl_3_): *δ* = 1.24, 1.52, 1.62 (3s, 9H, 3C*H*_3_); 1.93 (s, 4 h, C*H*_2_C*H*_2_); 3.74 (d, 2H, *J* = 8.0 Hz, C*H*_2_SO_2_); 4.96 (m, 1H, C*H*C=C(CH_3_)_2_); 5.12 (t, 1H, *J* = 7.8 Hz, C*H*CH_2_SO_2_); 7.46 (t, 2H, *J* = 7.8 Hz, C*H*_Ar_); 7.57 (t, 1H, *J* = 7.4 Hz, C*H*_Ar_); 7.80 ppm (d, 2H, *J* = 8.0 Hz, C*H*_Ar_); ^13^C-NMR (CDCl_3_): *δ* = 16.10, 17.65, 25.66 (3*C*H_3_); 26.13, 39.63 (*C*H_2_*C*H_2_); 56.04 (*C*H_2_SO_2_); 110.25 (*C*HCH_2_SO_2_); 123.39 (*C*HC=C(CH_3_)_2_); 128.52, 128.89, 133.49 (*C*H_Ar_); 132.04, 138.56, 146.34 ppm (2*C*=CH and *C*SO_2_); MS (ESI) *m/z*: 301.17 [M+Na]^+^.

*(2E,6E)-2,6-Dimethyl-8-(phenylsulfonyl)octa-2,6-dien-1-al* (**25**): Under argon and in the dark, SeO_2_ (8 mg, 0.07 mmol, 0.1 eq.), *t*-BuOOH (233 mg, 2.59 mmol, 3.6 eq.) and salicylic acid (4-hydroxybenzoic acid) (10 mg, 0.07 mmol, 0.1 eq.) were dissolved in CH_2_Cl_2_ (1 mL). A solution of compound **24** (200 mg, 0.72 mmol, 1 eq.) in CH_2_Cl_2_ (10 mL) was added at 0 °C. The ice bath was removed after 10 min and stirring was left for 24 h. The mixture was diluted in CH_2_Cl_2_, washed with a saturated solution of NaHCO_3_ then with water to neutralize tBuOOH and to eliminate HSeCO_3_. The organic layer was dried, filtered and concentrated *in vacuo*. Purification by chromatography on silica gel (EtOAc/petroleum ether 3:7) gave the aldehyde and the alcohol (50:50): *R*_f_ = 0.50 (EtOAc/petroleum ether 3:7); ^1^H-NMR (CDCl_3_): *δ* = 1.39, 1.70 (2s, 6H, 2C*H*_3_); 2.18 (t, 2H, *J* = 7.4 Hz, C*H*_2_CH_2_); 2.38 (q, 2H, *J* = 7.4 Hz, *J* = 15.0 Hz, C*H*_2_CH_2_); 3.81 (d, 2H, *J* = 8.0 Hz, C*H*_2_SO_2_); 5.22 (td, 1H, *J* = 1.2 Hz, *J* = 8.0 Hz, C*H*CH_2_SO_2_); 6.38 (td, 1H, *J* = 1.2 Hz, *J* = 7.2 Hz, C*H=C*CHO); 7.52 (t, 2H, *J* = 7.8 Hz, C*H*_Ar_); 7.63 (t, 1H, *J* = 7.6 Hz, C*H*_Ar_); 7.84 (d, 2H, *J* = 7.2 Hz, C*H*_Ar_); 9.36 ppm (s, 1H, C*H*O); ^13^C-NMR (CDCl_3_): *δ* = 9.16, 16.10 (2*C*H_3_); 26.78 (*C*H_2_CH_2_); 37.85 (*C*H_2_CH_2_); 55.82 (*C*H_2_SO_2_); 111.31 (*C*HCH_2_SO_2_); 128.26,129.00,133.62 (3*C*H_Ar_); 152.87 (*C*H=CCHO); 138.64, 139.62, 144.75 (2*C*=CH and *C*SO_2_); 194.97 ppm (*C*HO); MS (ESI) *m/z*: 315.17 [M+Na]^+^.

*(2E,6E)-2,6-Dimethyl-8-(phenylsulfonyl)octa-2,6-dien-1-ol* (**26**): To a solution containing compound **25** (8 g, 27.40 mmol, 1 eq.) in EtOH (80 mL) NaBH_4_ (1.04 g, 27.40 mmol, 1 eq.) was added in several portions. After 10 min at 0 °C, the mixture was diluted with CH_2_Cl_2_ and then washed with water. The organic layer was dried (Na_2_SO_4_), filtered and reduced under pressure. Purification by chromatography on silica gel (EtOAc/petroleum ether 1:1) gave a colourless oil (63% in alcohol, 2 steps): *R*_f_ = 0.30 (EtOAc/petroleum ether 3:7); ^1^H-NMR (CDCl_3_): *δ* = 1.37, 1.66 (2s, 6H, 2C*H*_3_); 2.08 (m, 4 h, C*H*_2_C*H*_2_); 3.80 (d, 2H, *J* = 7.6 Hz, C*H*_2_SO_2_); 3.99 (s, 2H, C*H*_2_OH); 5.19 (t, 1H, *J* = 7.4 Hz, C*H*CH_2_SO_2_); 5.33 (t, 1H, *J* = 6.0 Hz, C*H*=CCH_2_OH); 7.54 (t, 2H, *J* = 7.8 Hz, C*H*_Ar_); 7.64 (t, 1H, *J* = 7.4 Hz, C*H*_Ar_); 7.87 ppm (d, 2H, *J* = 7.6 Hz, C*H*_Ar_); ^13^C-NMR (CDCl_3_): *δ* = 13.68, 16.13 (2*C*H_3_); 25.39 (*C*H_2_CH_2_); 39.17 (*C*H_2_CH_2_); 56.00 (*C*H_2_SO_2_); 68.70 (*C*H_2_OH); 110.34 (*C*HCH_2_SO_2_); 124.63 (*C*H=CCH_2_OH); 128.37 (*C*H_Ar_); 129.02 (*C*H_Ar_); 133.58 (*C*H_Ar_); 135.54, 138.81, 146.11 ppm (2*C*=CH and *C*SO_2_); MS (ESI) *m/z*: 317.19 [M+Na]^+^.

S-(2E,6E)-2,6-Dimethyl-8-(phenylsulfonyl)octa-2,6-dien-1-yl ethanethioate (**27**): Firstly, compound **26** (4 g, 13.60 mmol, 1 eq.), CBr_4_ (5.41 g, 16.33 mmol, 1.2 eq.) and PPh_3_ (5 g, 19.05 mmol, 1.4 eq.) were reacted in CH_2_Cl_2_ (7 mL) at RT. After 2h, the mixture was reduced under pressure then purified by chromatography on silica gel (EtOAc/petroleum ether 2:3) to give a yellow oil (quantitative): *R*_f_ = 0.66 (EtOAc/petroleum ether5:5); ^1^H-NMR (CDCl_3_): *δ* = 1.33,1.73 (2s, 6H, 2C*H*_3_); 2.05 (m, 4 h, C*H*_2_C*H*_2_); 3.81 (d, 2H, *J* = 8.0 Hz, C*H*_2_SO_2_); 3.95 (s, 2H, C*H*_2_Br); 5.19 (t, 1H, *J* = 8.0 Hz, C*H*CH_2_SO_2_); 5.50 (m, 1H, C*H*=CCH_2_Br); 7.54 (t, 2H, *J* = 7.6 Hz, C*H*_Ar_); 7.64 (t, 1H, *J* = 7.4 Hz, C*H*_Ar_); 7.86 ppm (d, 2H, *J* = 7.2 Hz, C*H*_Ar_); ^13^C-NMR (CDCl_3_): *δ* = 14.65, 16.13 (2*C*H_3_); 26.29 (*C*H_2_CH_2_); 38.68 (*C*H_2_CH_2_); 41.42 (*C*H_2_Br); 55.95 (*C*H_2_SO_2_); 110.78 (*C*HCH_2_SO_2_); 128.35 (*C*H_Ar_); 128.98 (*C*H_Ar_); 129.99 (*C*H_2_Br); 133.56 (*C*H_Ar_); 132.63, 138.63, 145.57 ppm (2*C*=CH and *C*SO_2_); MS (ESI) *m/z*: 379.04 and 381.09 [M+Na]^+^, 394.94 and 397.06 [M+K]^+^.

Next, to a solution of the preceeding intermediate (5 g, 14.00 mmol, 1 eq.) in DMF (50 mL) KSAc (3.2 g, 28.00 mmol, 2 eq.) was added. After 1h at RT, the solution was diluted in CH_2_Cl_2_ and washed with water. The organic layer was dried, filtered and reduced under pressure and purification by chromatography on silica gel (EtOAc/petroleum ether 1:1) gave a brown oil (quantitative): *R*_f_ = 0.66 (EtOAc/petroleum ether 1:1); ^1^H-NMR (CDCl_3_): *δ* = 1.30, 1.60 (2s, 6H, 2C*H*_3_); 2.01 (m, 4 h, C*H*_2_C*H*_2_); 2.32 (s, 3H, COC*H*_3_); 3.51 (s, 2H, C*H*_2_S); 3.80 (d, 2H, *J* = 8.0 Hz, C*H*_2_SO_2_); 5.17 (t, 1H, *J* = 8.4 Hz, C*H*CH_2_SO_2_); 5.31 (m, 1H, C*H*=CH_2_S); 7.53 (t, 2H, *J* = 7.6 Hz, C*H*_Ar_); 7.63 (t, 1H, *J* = 7.4 Hz, C*H*_Ar_); 7.86 ppm (d, 2H, *J* = 7.2 Hz, C*H*_Ar_); ^13^C-NMR (CDCl_3_): *δ* = 15.14, 16.12 (2*C*H_3_); 26.18 (*C*H_2_CH_2_); 30.48 (CO*C*H_3_); 38.00 (*C*H_2_S); 39.09 (*C*H_2_CH_2_); 55.99 (*C*H_2_SO_2_); 110.59 (*C*HCH_2_SO_2_); 127.77 (*C*H=CCH_2_S); 128.47 (*C*H_Ar_); 128.94 (*C*H_Ar_); 133.52 (*C*H_Ar_); 131.01, 138.62, 145.85 ppm (2*C*=CH and *C*SO_2_); 195.55 (*C*O); MS (ESI) *m/z*: 375.67 [M+Na]^+^.

*(2Z,6E,10E)-12-(Acetylthio)-7,11-dimethyl-5-(phenylsulfonyl)dodeca-2,6,10-trien-1-yl 2,3,4-tri-O-acetyl-6-deoxy-6-azido-α-d-mannopyranoside* (**28**): Under argon, the solution of compound **27** (200 mg, 0.57 mmol, 1.3 eq.) dissolved in anhydrous THF (4 mL) was cooled to −78 °C and LiHMDS (550 µL) was slowly added. After 10 min, a solution containing compound **18** (200 mg, 0.43 mmol, 1 eq.) was introduced dropwise. The reaction was left at −78 °C under for 4 h then 18 h at RT. The mixture was diluted in CH_2_Cl_2_ then washed with water. The organic layer was dried, filtered and reduced under pressure and purification by chromatography on silica gel (EtOAc/petroleum ether 1:1) gave a colourless oil (15%): *R*_f_ = 0.33 (EtOAc/petroleum ether 1:1); 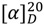 = +10.9 (*c* = 1.00 in chloroform); ^1^H-NMR (CDCl_3_): *δ* = 1.29, 1.59 (2s, 6H, 2CH=CC*H*_3_); 2.00 (m, 4 h, C*H*_2_C*H*_2_); 2.01, 2.09, 2.14, 2.31 (4s, 12H, 4COC*H*_3_); 3.50 (s, 2H, C*H*_2_S); 3.78 (m, 1H, C*H*SO_2_); 3.97 (m, 4 h, H_6_ and C*H*_2_CHSO_2_); 4.11 (m, 3H, *H*_5_ and C*H*_2_O); 4.64 (d, 1H, *J* = 1.2 Hz, *H*_1_); 4.74 (m, 2H, OCH_2_C*H*=C*H*); 5.11 (t, 1H, *J* = 10.2 Hz, *H*_4_); 5.20 (m, 1H, C*H*CHSO_2_); 5.30 (m, 2H, *H*_2_ and C*H*=CCH_2_S); 5.35 (dd, 1H, *J* = 10.4 Hz, *J* = 3.6 Hz, *H*_3_); 7.53 (m, 2H, C*H*_Ar_); 7.64 (m, 1H, C*H*_Ar_); 7.84 ppm (d, 2H, *J* = 7.2 Hz, C*H*_Ar_); ^13^C-NMR (CDCl_3_): *δ* = 15.11, 16.09 (2CH=C*C*H_3_); 20.58, 20.72, 20.79, 30.44 (4CO*C*H_3_); 26.15, 39.05 (*C*H_2_*C*H_2_); 37.96 (*C*H_2_S); 55.53 (*C*HSO_2_); 55.96, 59.43 (*C*_6_ and *C*HCHSO_2_); 62.24 (*C*H_2_O); 63.25 (*C*_5_); 66.10 (*C*_3_); 66.82 (*C*_4_); 70.31 (*C*_2_); 98.34, 98.65 (OCH_2_*C*H=*C*H); 99.35 (*C*_1_); 110.57 (*C*HCHSO_2_); 127.73 (*C*H=CCH_2_S); 128.43 (*C*H_Ar_); 128.91 (*C*H_Ar_); 131.96 (*C*H_Ar_); 133.49 (*C*H_Ar_); 130.97, 138.61, 145.81 (2*C*=CH and *C*SO_2_); 169.63, 169.73, 170.78 ppm (4*C*=O); MS (ESI) *m/z*: 737.01 [M+H]^+^, 758.98 [M+Na]^+^.

*(2Z,6E,10E)-12-(Acetylthio)-7,11-dimethyl-5-(phenylsulfonyl)dodeca-2,6,10-trien-1-yl 2,3,4-tri-O-acetyl-6-deoxy-α-d-heptomannopyranosiduronic acid* (**29**): The procedure described for compound **28** was applied to **22** and **27** to give compound **29** as a beige oil (17%): *R*_f_ = 0.16 (EtOAc/petroleum ether 1:1); 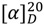 = +9.4 (*c* = 1.00 in chloroform); ^1^H-NMR (CDCl_3_): *δ* = 1.29, 1.58 (2s, 6H, 2CH=CC*H*_3_); 1.99 (m, 4 h, C*H*_2_C*H*_2_); 2.04, 2.07, 2.15, 2.31 (4s, 12H, 4COC*H*_3_); 3.50 (s, 2H, C*H*_2_S); 3.78 (m, 1H, C*H*SO_2_); 4.05 (m, 5 h, *H*_5_, C*H*_2_O and C*H*_2_CHSO_2_); 4.12 (dd, 1H, *J* = 2.4 Hz, *J* = 14.4 Hz, *H*_6a_); 4.27 (dd, 1H, *J* = 4.8 Hz, *J* = 12.4 Hz, *H*_6b_); 5.15 (t, 4 h, *J* = 8.0 Hz, C*H*=C*H*, 2C*H*=CCH_3_); 5.25 (m, 1H, *H*_2_); 5.32 (m, 2H, *H*_3_ and *H*_4_); 6.08 (d, 1H, *J* = 1.6 Hz, *H*_1_); 7.52 (t, 2H, *J* = 7.6 Hz, C*H*_Ar_); 7.63 (m, 1H, C*H*_Ar_); 7.84 ppm (d, 2H, *J* = 7.6 Hz, C*H*_Ar_); ^13^C-NMR (CDCl_3_): *δ* = 15.02, 15.99 (2CH=C*C*H_3_); 20.50, 20.60, 20.70 (4CO*C*H_3_); 26.05, 38.95 (*C*H_2_*C*H_2_); 37.86 (CH_2_S); 55.85 (*C*SO_2_); 61.92 (*C*_6_, *C*H_2_O and *C*H_2_CHSO_2_); 65.33, 68.57 (*C*_3_ and *C*_4_); 68.16 (*C*_2_); 70.42 (*C*_5_); 90.41 (*C*_1_); 110.49, 126.98 (2*C*H=CCH_3_); 110.81 (*C*H=*C*H); 127.44–133.42 (5*C*H_Ar_); 138.48, 145.70, 146.84 (2*C*=CH and *C*SO_2_); 169.36, 169.56, 168.80, 170.44 (4*C*=O); 195.37 ppm (*C*O_2_H); MS (ESI) *m/z*: 740.05 [M+H]^+^, 761.99 [M+Na]^+^.

*(2Z,6E,10E)-12-Mercapto-7,11-dimethyl-5-(phenylsulfonyl)dodeca-2,6,10-trien-1-yl-6-deoxy-6-azido-α-D-mannopyranoside* (**30**): The procedure described for compound **3** was applied to **2** to give compound **30** as a white oil (97%): *R*_f_ = 0.34 (CH_2_Cl_2_/MeOH 4:1); ^1^H-NMR (CDCl_3_): *δ* = 1.61, 1.66 (2s, 6H, 2C*H*_3_); 2.02, 2.11 (2m, 4 h, C*H*_2_C*H*_2_); 3.49 (m, 1H, *H*_5_); 3.61 (t, 1H, *J* = 9.4 Hz, *H*_4_); 3.67 (dd, 1H, *J* = 3.2 Hz, *J* = 9.2 Hz, *H*_3_); 3.72 (dd, 1H, *J* = 5.6 Hz, *J* = 12.0 Hz, *H*_6a_); 3.79 (q, 1H, *J* = 1.6 Hz, *H*_2_); 3.83 (dd, 1H, *J* = 2.4 Hz, *J* = 12.0 Hz, *H*_6b_); 4.08 (d, 4 h, *J* = 6.4 Hz, C*H*_2_O and C*H*_2_S); 4.64 (d, 1H, *J* = 1.6 Hz, *H*_1_); 5.11 (m, 3H, 3C*H*=C); 5.35 ppm (m, 3H, 3C*H*=C); ^13^C-NMR (CDCl_3_): *δ* = 16.27, 25.89 (2*C*H_3_); 27.52 (*C*H_2_CH_2_); 40.72 (*C*H_2_CH_2_); 59.45 (*C*H_2_O and *C*H_2_S); 62.83 (*C*_6_); 68.57 (*C*_4_); 72.09 (*C*_2_); 72.59 (*C*_3_); 74.42 (*C*_5_); 102.71 (*C*_1_); 124.92, 125.14, 129.95, 130.07, 133.05, 133.15 (6*C*H=C); 132.44, 139.40 ppm (2*C*=CH); MS (ESI) *m/z*: 426.12 [M+H]^+^, 448.24 [M+Na]^+^.

*(2Z,6E,10E)-12-Mercapto-7,11-dimethyl-5-(phenylsulfonyl)dodeca-2,6,10-trien-1-yl-6-deoxy-α-d-heptomannopyranosiduronic acid* (**31**): The procedure described for compound **3** was applied to **29** to give compound **31** as a white oil (97%): *R*_f_ = 0.50 (*i*-PrOH/NH_4_OH 1:1); 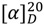 = +4.4 (*c* = 1.00 in chloroform); ^1^H-NMR (CDCl_3_): *δ* = 1.30, 1.55 (2s, 6H, 2C*H*_3_); 1.99 (m, 4 h, C*H*_2_C*H*_2_); 3.50 (s, 2H, C*H*_2_S); 3.76 (m, 1H, C*H*=CH); 4.03 (m, 5 h, *H*_5_, C*H*_2_O and C*H*=CH); 4.12 (dd, 1H, *J* = 2.4 Hz, *J* = 14.4 Hz, *H*_6a_); 4.27 (dd, 1H, *J* = 4.8 Hz, *J* = 12.4 Hz, *H*_6b_); 5.15 (t, 4 h, *J* = 8.0 Hz, 2C*H*=CH, 2C*H*=C); 5.25 (m, 1H, *H*_2_); 5.32 (m, 2H, *H*_3_ and *H*_4_); 6.08 ppm (d, 1H, *J* = 1.6 Hz, *H*_1_); ^13^C-NMR (CDCl_3_): δ = 15.02, 15.99 (2*C*H_3_); 26.05, 38.95 (*C*H_2_*C*H_2_); 37.86 (*C*H_2_S); 55.85 (*C*H=CH); 61.92 (C_6_, *C*H_2_O and*C*H=CH); 65.33, 68.57 (*C*_3_ and *C*_4_); 68.16 (*C*_2_); 70.42 (*C*_5_); 90.41 (*C*_1_); 110.49, 126.98 (2*C*H=C); 110.81 (2*C*H=CH); 138.48, 145.70 (2*C*=CH); 195.37 ppm (*C*O_2_H); MS (ESI) *m/z*: 471.76 [M+H]^+^, 493.78 [M+Na]^+^.

*6-Deoxy-6-azido-2,3,4-tri-O-acetyl-α-d-mannopyranosyl bromide* (**32**): A solution of hydrobromic acid (5.7 M in acetic acid, 2.35 mL,13.40 mmol, 25 eq.) was added to a solution of acetic anhydride (1 mL) and compound **17** (200 mg, 0.54 mmol, 1 eq.). After 16 h at RT, the mixture was diluted in CH_2_Cl_2_ and washed with a saturated solution of NaHCO_3_ until basic pH. The aqueous phase was extracted with CH_2_Cl_2_ (3 times). The organic layers were assembled, then washed with a saturated solution of NaCl, dried, filtered and concentrated *in vacuo*. The obtained colourless oil was used without purification (quantitative): *R*_f_ = 0.56 (petroleum ether/Et_2_O 3:7); 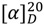 = +96.1 (*c* = 1.00 in chloroform); ^1^H-NMR (CDCl_3_): *δ* = 2.01, 2.10, 2.17 (3s, 9H, 3C*H*_3_); 3.21 (dd, 1H, *J* = 6.6 Hz, *J* = 11.4 Hz, *H*_6a_); 3.35 (dd, 1H, *J* = 2.9 Hz, *J* = 11.4 Hz, *H*_6b_); 3.94–3.99 (m, 1H, *H*_5_); 5.27 (t, 1H, *J* = 10.0 Hz, *H*_4_); 5.41 (dd, 1H, *J* = 1.4 Hz, *J* = 3.3 Hz, *H*_2_); 5.71 (dd, 1H, *J* = 3.4 Hz, *J* = 10.0 Hz, *H*_3_); 6.3 ppm (d, 1H, *J* = 1.1 Hz, *H*_1_); ^13^C-NMR (CDCl_3_): δ = 2.46 (*C*_6_); 20.60, 20.73, 20.78 (3*C*H_3_); 67.66 (*C*_3_); 69.49 (*C*_4_); 72.21 (*C*_2_); 73.45 (*C*_5_); 82.46 (*C*_1_); 169.58, 169.64, 169.72 (3*C*=O); MS (ESI) *m/z*: 393.98, 395.40 [M+H]^+^.

*6-Deoxy-6-azido-2,3,4-tri-O-acetyl-1-thio-β-d-mannopyranose* (**34**): A solution of compound **32** (100 mg, 0.25 mmol, 1 eq.) and thiourea (25 mg, 0.33 mmol) in acetone (2 mL) was stirred under reflux for 20 h. The reaction was cooled to room temperature. The solvent was removed under reduced pressure to give the isothiouronium salt as a white solid. K_2_S_2_O_5_ (85 mg, 0.38 mmol) was added to a suspension of this salt in CHCl_3_/H_2_O (1/1 *v/v*) (3 mL). After stirring under reflux for 5 h, the solution was cooled to RT, the CHCl_3_ layer was separated and the aqueous layer was extracted with CH_2_Cl_2_. The combined organic layers were dried and concentrated under reduced pressure. Purification by chromatography onsilica gel (EtOAc/petroleum ether 1:1) gave a colourless oil (50%): *R*_f_ = 0.56 (CH_2_Cl_2_/Et_2_O 8:2); ^1^H- NMR (CDCl_3_): *δ* = 2.00, 2.06, 2.19 (3s, 9H, 3C*H*_3_); 3.32 (m, 2H, *H*_6_); 4.17 (ddd, 1H, *J* = 4.1 Hz, *J* = 5.4 Hz, *J* = 9.5 Hz, *H*_5_); 4.56 (d, 1H, *J* = 4.1 Hz, *H*_1_); 5.22 (m, 2H, *H*_2_ et *H*_4_); 5.39 ppm (dd, 1H, *J* = 3.2 Hz, *J* = 10.0 Hz, *H*_3_); ^13^C-NMR (CDCl_3_): *δ* = 20.6, 20.6, 20.8 (3*C*H_3_); 51.1 (*C*_6_); 67.2 (*C*_4_); 68.6 (*C*_3_); 69.5 (*C*_5_); 70.1 (*C*_2_); 91.8 (*C*_1_); 169.9, 170.1, 170.3 (3*C*=O); MS (ESI) *m/z*: 370.44 [M+Na]^+^.

*6-Deoxy-2,3,4-tri-O-acetyl-α-d-heptomannopyranuronosyl bromide* (**33**): The procedure described for compound **32** was applied to **21** to give compound **33** as a white oil (quant): *R*_f_ = 0.22 (petroleum ether/EtOAc 1:1); ^1^H-NMR (CDCl_3_): *δ* = 1.97, 2.05, 2.12 (3s, 9H, C*H*_3_); 1.96–2.21 (m, 2H, *H*_6_); 3.91 (td, 1H, *J* = 2.6 Hz, *J* = 10.0 Hz, *H*_5_); 5.13 (t, 1H, *J* = 10.0 Hz, *H*_4_); 5.20 (dd, 1H, *J* = 1.9 Hz, *J* = 3.5 Hz, *H*_2_); 5.27 (dd,1H, *J* = 3.5 Hz, *J* = 10.0 Hz, *H*_3_); 5.94 ppm (d, 1H, *J* = 1.8 Hz, *H*_1_); ^13^C-NMR (CDCl_3_): *δ* = 20.60, 20.75 (3*C*H_3_); 30.02 (*C*_6_); 68.47 (*C*_2_); 68.74 (*C*_3_); 68.97 (*C*_4_); 69.24 (*C*_5_); 90.11 (*C*_1_); 168.09, 169.00, 169.33, 169.75 (3*C*=O et *C*O_2_H); MS (ESI) *m/z*: 419.67, 421.28 [M+Na]^+^.

*6-Deoxy-2,3,4-tri-O-acetyl-1-thio-β-d-heptomannopyranuronic acid* (**35**): The procedure described for compound **34** was applied to **33** to give compound **35** as a white oil (48%): *R*_f_ = 0.20 (CH_2_Cl_2_/MeOH 4:1); ^1^H-NMR (CDCl_3_): *δ* = 1.99, 2.06, 2.14 (3s, 9H, C*H*_3_); 1.95–2.26 (m, 2H, *H*_6_); 4.01 (td, 1H, *J* = 2.6 Hz, *J* = 10.0 Hz, *H*_5_); 5.11 (t, 1H, *J* = 10.0 Hz, *H*_4_); 5.12 (d, 1H, *J* = 3.2 Hz, *H*_1_); 5.23 (dd, 1H, *J* = 1.7 Hz, J = 3.4 Hz, *H*_2_); 5.35 ppm (dd,1H, *J* = 3.4 Hz, *J* = 10.0 Hz, *H*_3_); ^13^C-NMR (CDCl_3_): *δ* = 20.72, 20.88, 20.93 (3*C*H_3_); 30.32 (*C*_6_); 67.46 (*C*_5_); 68.88 (*C*_3_); 69.46 (*C*_4_); 70.11 (*C*_2_); 92.03 (*C*_1_); 169.40, 167.55, 170.08, 170.13 (3*C*=O et *C*O_2_H); MS (ESI) *m/z*: 373.28 [M+Na]^+^.

*2-{2-[2-(Allyloxy)ethoxy]ethoxy}ethanol* (**36**): The procedure described for compound **6** was applied to triethylene glycol and 3-bromopropene to give compound **36** as a red-orange oil (85%): *R*_f_ = 0.34 (CH_2_Cl_2_/MeOH 9:1); ^1^H-NMR (CDCl_3_): *δ* = 3.57–3.71 (m, 12H, 6C*H*_2_O); 4.00 (m, 2H, C*H*_2_CH=CH_2_); 5.16 (dd, 1H, *J* = 1.6 Hz, *J* = 10.4 Hz, C*H*_2_ = CH); 5.25 (dd, 1H, *J* = 1.6 Hz, *J* = 17.2 Hz, C*H*_2_=CH); 5.89 ppm (m, 1H, C*H*=CH_2_); ^13^C-NMR (CDCl_3_): *δ* = 61.66–72.57 (7*C*H_2_O); 117.30 (*C*H_2_=CH); 134.59 ppm (*C*H=CH_2_); MS (ESI) *m/z*: 213.22 [M+Na]^+^, 229.17 [M+K]^+^.

*3-{2-[2-(2-Hydroxyethoxy)ethoxy]ethoxy}propyl2,3,4-tri-O-acetyl-6-deoxy-6-azido-1-thio-β-d-manno-pyranoside* (**37**): Under argon, compound **36** (36 mg, 0.19 mmol) and AIBN (0.29 mmol, 1.5 eq.) were added to a solution of compound **34** (200 mg, 0.58 mmol) in dioxane degassed under argon (12 mL). After 3 h under ultrasound (amplitude of 20%, pulse on 0.2 s, pulse off 0.2 s), the mixture was reduced under pressure then purified by chromatography on silica gel (EtOAc/petroleum ether 7:3 to 10:0) to give a colourless oil (79%) *R*_f_ = 0.38 (EtOAc); 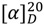 = +43.8 (*c* = 1.00 in chloroform); ^1^H-NMR (CDCl_3_): *δ* = 1.98 (s, 3H, C*H*_3_); 2.03 (m, 5 h, C*H*_2_CH_2_S and C*H*_3_); 2.14 (s, 3H, C*H*_3_); 3.27 (m, 5 h, *H*_6a_, C*H*_2_S and C*H*_2_(CH_2_)_2_S); 3.35 (dd, 1H, *J* = 6.4 Hz, *J* = 13.2 Hz, *H*_6b_); 3.59–3.72 (m, 12H, 6C*H*_2_O); 4.01 (m, 1H, *H*_5_); 4.87 (d, 1H, *J* = 7.4 Hz, *H*_1_); 5.22 (t, 1H, *J* = 10.0 Hz, *H*_4_); 5.25 (dd, 1H, *J* = 1.6 Hz, *J* = 3.6 Hz, *H*_2_); 5.35 ppm (dd, 1H, *J* = 3.2 Hz, *J* = 10.0 Hz, *H*_3_); ^13^C-NMR (CDCl_3_): *δ* = 20.65, 20.69, 20.83 (3*C*H_3_); 27.98 (*C*H_2_S); 35.59 (*C*H_2_CH_2_S); 58.73 (*CH*_2_(CH_2_)_2_S); 51.05 (*C*_6_); 61.62–72.51 (*C*H_2_O); 67.16 (*C*_4_); 68.80 (*C*_3_); 69.48 (*C*_2_); 69.93 (*C*_5_); 97.42 (*C*_1_); 169.82, 169.95, 170.08 ppm (3*C*=O); MS(ESI) *m/z*: 560.12 [M+Na]^+^.

*3-{2-[2-(2-Hydroxyethoxy)ethoxy]ethoxy}propyl2,3,4-tri-O-ace-tyl-6-deoxy-6-azido-1-thio-β-d-hepto-mannopyranosiduronic acid* (**38**): The procedure described for compound **37** was applied to **35** and **36** to give compound **38** as a beige oil (80%): *R*_f_ = 0.08 (EtOAc); 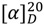 = +40.0 (*c* = 1.00 in chloroform); ^1^H-NMR (CDCl_3_): *δ* = 1.87 (qt, 2H, *J* = 6.8 Hz, C*H*_2_CH_2_S); 1.95, 2.06, 2.12 (3s, 12H, 4C*H*_3_); 2.69 (m, 2H, C*H*_2_S); 3.51 (t, 2H, *J* = 6.0 Hz, C*H*_2_(CH_2_)_2_S); 3.54–3.69 (m, 12H, 6C*H*_2_O); 4.05 (dd, 1H, *J* = 2.2 Hz, *J* = 12.2 Hz, *H*_6a_); 4.27 (dd, 1H, *J* = 5.4 Hz, *J* = 12.2 Hz, *H*_6b_); 4.34 (m, 1H, *H*_5_); 4.80 (d, 1H, *J* = 7.4 Hz, *H*_1_); 5.23 (m, 2H, *H*_3_ et *H*_4_); 5.30 ppm (dd, 1H, *J* = 1.6 Hz, *J* = 3.2 Hz, *H*_2_); ^13^C-NMR (CDCl_3_): *δ* = 19.62, 19.69, 19.91 (3*C*H_3_); 27.19 (*C*H_2_S); 28.41 (*C*H_2_CH_2_S); 60.69 (*C*H_2_(CH_2_)_2_S); 61.41 (*C*_6_); 65.31 (*C*_3_); 67.96 (*C*_5_); 68.45 (*C*_4_); 68.26–71.53 (7*C*H_2_O); 70.14 (*C*_2_); 81.64 (*C*_1_); 168.72, 168.80, 168.99, 169.63 ppm (3*C*=O et *C*O_2_H); MS (ESI) *m/z*: 563.76 [M+Na]^+^.

*3-{2-[2-(2-Mercaptoethoxy)ethoxy]ethoxy}propyl 6-deoxy-6-azido-1-thio-β-d-mannopyranoside* (**39**): The procedures described for compounds **27** then **3** were applied to **37** to give compound **39** as a beige oil: *R*_f_ = 0.21 (CH_2_Cl_2_/MeOH 4:1); 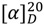 = +40.0 (*c* = 1.00 in chloroform); ^1^H-NMR (CD_3_OD): *δ* = 1.95 (m, 2H, C*H*_2_CH_2_SC); 2.88 (t, 2H, *J* = 6.6 Hz, C*H*_2_SH); 3.26 (m, 4 h, C*H*_2_SC and C*H*_2_(CH_2_)_2_SC); 3.40 (dd, 1H, *J* = 7.0 Hz, *J* = 13.0 Hz, *H*_6a_); 3.55 (m, 2H, *H*_5_ and *H*_6a_); 3.58–3.69 (m, 10H, *H*_3,4_ and 4C*H*_2_O); 3.72 (t, 2H, *J* = 6.4 Hz, C*H*_2_CH_2_SH); 3.80 (dd, 1H, *J* = 1.6 Hz, *J* = 3.2 Hz, *H*_2_); 4.77 ppm (d, 1H, *J* = 1.6 Hz, *H*_1_); ^13^C-NMR (CD_3_OD): *δ* = 28.02 (*C*H_2_SC); 37.42 (*C*H_2_CH_2_SC); 39.51 (*C*H_2_SH); 57.92 (*C*H_2_(CH_2_)_2_SC); 53.02 (*C*_6_); 62.55–71.65 (5*C*H_2_O); 69.52, 72.01, 72.36 (*C*_3_, *C*_4_ and *C*_5_); 73.86 (*C*_2_); 101.87 ppm (*C*_1_); MS (ESI) *m/z*: 428.73 [M+H]^+^, 460.57 [M+Na]^+^.

*3-{2-[2-(2-Mercaptoethoxy)ethoxy]ethoxy}propyl 6-deoxy-1-thio-β-d-heptomannopyranosiduronic acid* (**40**): The procedures described for compounds **27** then **3** were applied to **38** to give compound **40** as a beige oil: *R*_f_ = 0.18 (*i*-PrOH/NH_4_OH 1:1); 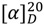 = +32.0 (*c* = 1.05 in chloroform); ^1^H-NMR (CD_3_OD): *δ* = 1.92 (qt, 2H, *J* = 6.4 Hz, C*H*_2_CH_2_SC); 2.73 (m, 2H, C*H*_2_SC); 3.09 (t, 2H, *J* = 6.6 Hz, C*H*_2_(CH_2_)_2_SC); 3.53 (t, 2H, *J* = 6.0 Hz, C*H*_2_SH); 3.55–3.62 (m, 10H, 5C*H*_2_O); 4.08 (dd, 1H, *J* = 2.2 Hz, *J* = 12.2 Hz, *H*_6a_); 4.30 (dd, 1H, *J* = 5.4 Hz, *J* = 12.2 Hz, *H*_6b_); 4.37 (m, 1H, *H*_5_); 5.21 (m, 2H, *H*_1_, *H*_3_ and *H*_4_); 5.31 (dd, 1H, *J* = 1.6 Hz, *J* = 3.2 Hz, *H*_2_); ^13^C-NMR (CD_3_OD): *δ* = 27.22 (*C*H_2_SC); 27.82 (*C*H_2_CH_2_SC); 28.48 (*C*H_2_(CH_2_)_2_SC); 61.41 (*C*_6_); 65.32 (*C*_3_); 67.96 (*C*_5_); 68.44 (*C*_4_); 68.28–69.59 (5*C*H_2_O and *C*H_2_SH); 70.14 (*C*_2_); 81.65 (*C*_1_);168.90 (*C*O_2_H); MS (ESI) *m/z*: 431.59 [M+H]^+^, 463.64 [M+Na]^+^.

### 3.2. Preparation of Citrate-Reduced Gold Nanoparticles

Hydrogen tetrachloroaurate trihydrate (60 mg, 0.15 mmol) was dissolved in water (250 mL) to give a pale-yellow solution. A second solution with sodium citrate (150 mg, 0.58 mmol) dissolved in water (10 mL) was prepared. Both solutions were heated to 60 °C for 10 min then the sodium citrate solution was rapidly added to the gold solution. The temperature was then increased to 120 °C with continuous stirring for 2 h 30 min. A deep-red solution was formed. The solution was warmed to RT and each thiol-derivatized carbohydrate (50 mg) dissolved in methanol (1 mL) was added to freshly prepared citrate-reduced gold nanoparticles. Self-assembly was facilitated by leaving the solution under stirring for 48 h. To remove any unbound carbohydrate, the solution was diluted with brine to precipitate nano-objects and left to rest over night. Then the supernatant was removed and nanoparticles were resuspended in water. They were then centrifuged for 30 min at 14,000 rpm. The centrifugation step was repeated three times to ensure complete removal of any unbound carbohydrate.

### 3.3. CAM Assays

**Figure 2:**
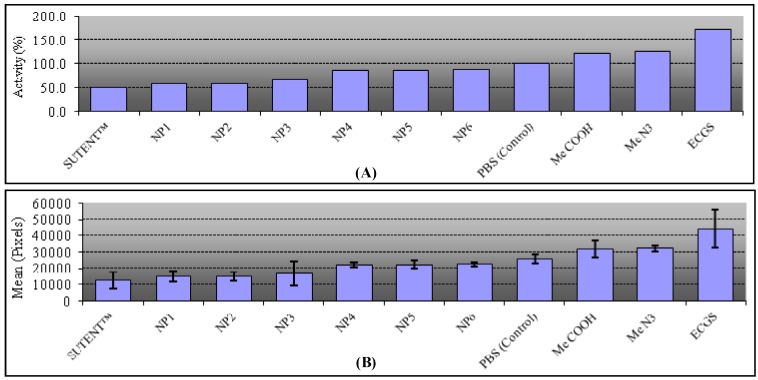
(**A**) CAM activity in percentage compared to PBS (control); (**B**) CAM activity in pixels (Image J) compared to PBS (control).

Paper discs were saturated with a phosphate buffer saline dispersion of coated AuNPs (60 mg/mL) in PBS or a control (phosphate buffer saline) and then deposited on chorioallantoic membranes of 7-day-old chicken embryos for 4 days in ovo at 38 °C. Sutent^®^ (sunitinib, a non-proteic inhibitor) and ECGS (endothelial cell growth supplement) were used at 60 mg/mL as negative and positive stimuli, respectively. Quantification of the angiogenic response was carried out by measuring the area of neo-vascularization on each particular membrane (Figure 2). The vascularization was evaluated using Image J software, and are given in pixels compared to phosphate buffer saline (PBS, control).

PBS: 25790.5+/−2534, *n =* 4 (100%); Sutent^®^: 12765+/−4913.5, *p* = 0.00003, *n* = 10; NP1: 14944.2+/−3348.2, *p* = 0.00044, *n* = 5; NP2: 15169+/−2527.2, *p* = 0.00027, *n* = 5; NP3: 16813+/−7274.4, *p* = 0.01444, *n* = 6; NP4: 22276.2+/−1549.8, *p* = 0.0309, *n* = 5; NP5: 22463.2+/−2210.6, *p* = 0.0416, *n* = 5; NP6: 22657.3+/−1215, *p* = 0.04239, *n* = 4; Uronic (methyl 6,7-dideoxy-α-D-mannopyranoside) acid (MeCOOH): 31758+/−4919.8, *p* = 0.0449, *n* = 4; 6-deoxy-6-azido-**α**-D-methylmannopyranoside (MeN3): 32348+/−1805.5, *p* = 0.00350, *n* = 4; ECGS: 44455.8+/−11955.3, *p* = 0.00138, *n* = 8.

## 4. Conclusions

We have reported the synthesis of a series of gold glyconanoparticles bearing diverse M6P neoglycoconjugates. These M6P analogues were synthesized either by Huisgen cycloaddition, by the Julia olefination, or by thiol-ene coupling. The thiol-ene reaction strategy under ultrasound activation proved to be the most efficient in terms of yields and ease of implementation. The angiogenic activities of the AuNPs have been tested by the CAM assay, and all possess angiogenic activities via the M6P receptor with no apparent toxicity. The results of this study have allowed us: (a) to demonstrate that the activity is not dependent of the structure of the linker between the nanoparticles and the carbohydrate and (b) to identify the inhibitory multivalent effect of M6P derivatives on gold surfaces compared with the corresponding monomeric activators. Although our biological results are obviously in a preliminary stage, the work described herein is valuable in that it provides synthetic access to some potentially useful multi-functional and biologically active systems.
